# Genome-wide and phenome-wide analysis of ideal cardiovascular health in the VA Million Veteran Program

**DOI:** 10.1371/journal.pone.0267900

**Published:** 2022-05-25

**Authors:** Rose D. L. Huang, Xuan-Mai T. Nguyen, Gina M. Peloso, Mark Trinder, Daniel C. Posner, Krishna G. Aragam, Yuk-Lam Ho, Julie A. Lynch, Scott M. Damrauer, Kyong-Mi Chang, Philip S. Tsao, Pradeep Natarajan, Themistocles Assimes, J. Michael Gaziano, Luc Djousse, Kelly Cho, Peter W. F. Wilson, Jennifer E. Huffman, Christopher J. O’Donnell, on behalf of the Veterans Affairs’ Million Veteran Program

**Affiliations:** 1 Center for Population Genomics, Massachusetts Veterans Epidemiology Research and Information Center (MAVERIC), VA Boston Healthcare System, Boston, Massachusetts, United States of America; 2 MAVERIC, VA Boston Healthcare System, Boston, Massachusetts, United States of America; 3 Carle Illinois College of Medicine, University of Illinois at Urbana-Champaign, Champaign, Illinois, United States of America; 4 Department of Biostatistics, Boston University School of Public Health, Boston, Massachusetts, United States of America; 5 Centre for Heart Lung Innovation, University of British Columbia, Vancouver, Canada; 6 Program in Medical and Population Genetics and the Cardiovascular Disease Initiative, Broad Institute of MIT and Harvard, Cambridge, Massachusetts, United States of America; 7 Cardiovascular Research Center, Massachusetts General Hospital, Harvard Medical School, Boston, Massachusetts, United States of America; 8 VA Informatics & Computing Infrastructure, VA Salt Lake City Health Care System, Salt Lake City, Utah, United States of America; 9 College of Nursing & Health Sciences, University of Massachusetts Boston, Boston, Massachusetts, United States of America; 10 Corporal Michael Crescenz VA Medical Center, Philadelphia, Pennsylvania, United States of America; 11 Department of Surgery, Perelman School of Medicine, University of Pennsylvania, Philadelphia, Pennsylvania, United States of America; 12 Department of Medicine, Perelman School of Medicine, University of Pennsylvania, Philadelphia, Pennsylvania, United States of America; 13 VA Palo Alto Health Care System, Palo Alto, California, United States of America; 14 Department of Medicine, Stanford University School of Medicine, Stanford, California, United States of America; 15 Department of Medicine, Harvard Medical School, Boston, Massachusetts, United States of America; 16 Division of Aging, Department of Medicine, Brigham and Women’s Hospital, Boston, Massachusetts, United States of America; 17 Atlanta VA Medical Center, Decatur, Georgia, United States of America; 18 Emory University School of Medicine, Atlanta, Georgia, United States of America; 19 Heart & Vascular Center, Brigham and Women’s Hospital, Boston, Massachusetts, United States of America; Johns Hopkins University, UNITED STATES

## Abstract

**Background:**

Genetic studies may help identify causal pathways; therefore, we sought to identify genetic determinants of ideal CVH and their association with CVD outcomes in the multi-population Veteran Administration Million Veteran Program.

**Methods:**

An ideal health score (IHS) was calculated from 3 clinical factors (blood pressure, total cholesterol, and blood glucose levels) and 3 behavioral factors (smoking status, physical activity, and BMI), ascertained at baseline. Multi-population genome-wide association study (GWAS) was performed on IHS and binary ideal health using linear and logistic regression, respectively. Using the genome-wide significant SNPs from the IHS GWAS, we created a weighted IHS polygenic risk score (PRS_IHS_) which was used (i) to conduct a phenome-wide association study (PheWAS) of associations between PRS_IHS_ and ICD-9 phenotypes and (ii) to further test for associations with mortality and selected CVD outcomes using logistic and Cox regression and, as an instrumental variable, in Mendelian Randomization.

**Results:**

The discovery and replication cohorts consisted of 142,404 (119,129 European American (EUR); 16,495 African American (AFR)), and 45,766 (37,646 EUR; 5,366 AFR) participants, respectively. The mean age was 65.8 years (SD = 11.2) and 92.7% were male. Overall, 4.2% exhibited ideal CVH based on the clinical and behavioral factors. In the multi-population meta-analysis, variants at 17 loci were associated with IHS and each had known GWAS associations with multiple components of the IHS. PheWAS analysis in 456,026 participants showed that increased PRS_IHS_ was associated with a lower odds ratio for many CVD outcomes and risk factors. Both IHS and PRS_IHS_ measures of ideal CVH were associated with significantly less CVD outcomes and CVD mortality.

**Conclusion:**

A set of high interest genetic variants contribute to the presence of ideal CVH in a multi-ethnic cohort of US Veterans. Genetically influenced ideal CVH is associated with lower odds of CVD outcomes and mortality.

## Introduction

In its 2020 Strategic Impact Goals, the American Heart Association (AHA) advanced the concept of ideal cardiovascular health (CVH), which focused on optimal levels of seven clinical and behavioral “health factors” associated with lower risk of cardiovascular disease (CVD). The AHA “Life’s Simple 7” (LS7) metrics for ideal CVH include three clinical health factors (total cholesterol, blood pressure, and blood glucose levels) and four behavioral health factors (body mass index (BMI), smoking status, diet quality, and physical activity) [1]. Strong evidence from recent observational studies indicates that the presence of multiple ideal CVH factors is associated with lower all-cause mortality, lower CVD mortality, and greater longevity [2–4]. However, several recent studies have documented a low prevalence of ideal CVH in diverse US populations and geographic settings, including an increasing prevalence of obesity [5–8], highlighting the urgent need for strategies to improve CVH.

Individual interventions on most of the individual health factors have resulted in net benefit. However, prior attempts to simultaneously modify multiple major CVD risk factors have had limited success. In the Multiple Risk Factor Intervention Trial, a randomized primary prevention trial conducted nearly four decades ago, randomization to simultaneously treat hypertension and elevated dietary cholesterol and reduce cigarette smoking did not result in a net significant benefit, although there was a longer-term mortality benefit in middle-aged men [9]. While the totality of evidence from randomized trials on primary prevention of CVD using multiple risk factor interventions does not provide evidence of a net mortality benefit, the trials were largely conducted decades ago with older interventions on some but not all risk factors [10, 11]. Better understanding of the mechanisms by which ideal CVH can be achieved through a combination of genetic, behavioral, and environmental factors may identify more precise CVH-specific strategies for modifiable risk factors, new drug or behavioral targets for modifying risk factors and/or identify key pathways or master regulators of CVH.

While numerous studies have examined the contribution of genetic factors to the occurrence of clinically apparent CVD and of individual CVD risk factors [12–15] that are components of ideal CVH, data are limited on the genetic basis of ideal CVH. In a genome-wide association study (GWAS) of ideal CVH in 11,708 men and women of European ancestry in the Cohorts for Heart and Aging Research in Genetic Epidemiology (CHARGE) consortium, a single genetic locus in the *APOC1/APOE* region was significantly associated with binary clinical ideal CVH based on cholesterol, blood pressure, and plasma glucose levels [16].

We conducted a GWAS to investigate the genetic determinants of ideal CVH in a large, diverse mega-biobank cohort recruited in the recent decade, the United States (US) Department of Veterans Affairs (VA) Million Veteran Program (MVP) [17]. The prevalence of ideal CVH among the MVP participants is low but in the same range as recent low prevalence estimates in other US-based multi-ethnic cohorts [18]. We hypothesized that we could identify genetic variants that contribute to ideal CVH and that may be protective against CVD in the multi-ethnic MVP cohort.

## Methods

### Study participants

Participants in MVP were recruited from more than 60 VA Medical Centers across the US beginning in 2011. MVP incorporated data from biospecimens, surveys, and electronic health records (EHR), which included clinical laboratory measurements, diagnostic imaging reports, Current Procedural Terminology (CPT) procedure codes, and International Classification of Diseases (ICD 9/10) diagnosis and procedure codes. MVP was approved by the VA Central Institutional Review Board and conformed to the Declaration of Helsinki principles. All MVP study participants provided informed written consent. Additional details of the MVP study protocol have been previously described [17]. The MVP participant populations used for each analysis are summarized in **S1 Table of** S1 File and described below.

### Ideal cardiovascular health phenotype

LS7 clinical components (blood pressure, blood glucose, and total cholesterol) were derived from the EHR using the value closest to enrollment date (within one year before or after study enrollment). Since fasting status could not be confirmed for more than half of our participants, some non-fasting plasma glucose values may have been used. Self-reported MVP baseline and lifestyle surveys were used to determine the LS7 behavioral components (BMI, physical activity, and smoking status) [18]. LS7 components were categorized as 0 = poor, 1 = intermediate, and 2 = ideal health based on the LS7 CVH factor criteria established by AHA (**S2 Table of** S1 File) [1]. Ideal diet was not considered since only 0.4% of participants fell into the ideal diet category. Methods for the measurement of each LS7 component and adaptations made in MVP to the AHA classifications have been reported elsewhere and are summarized in the **Supplemental Methods of** S2 File [18].

We calculated the ideal health score (IHS), which ranged from 0 to 12, by adding the individual component scores for each of the three clinical components and the three behavioral components. A binary variable for ideal health (BIH) was defined as having an overall IHS of 9 or greater. We also calculated a separate clinical IHS and a behavioral IHS using only the clinical or behavioral components, respectively, with both scores ranging from 0 to 6. Clinical BIH was defined by a clinical IHS of greater than or equal to 5 and behavioral BIH was defined by a behavioral IHS of greater than or equal to 4. The dichotomous cut-offs were established based on the proportion of cases (to optimize power) and the heterogeneity (to minimize the error rate).

### Genetic data

A customized Affymetrix Axiom Biobank Array containing 723,305 DNA sequence variants was used for genotyping MVP participant DNA extracted from whole blood. The array included tag SNPs validated for diseases and biomarkers of clinical relevance in diverse ethnic groups. Detailed information on quality-control measures that removed low-quality samples and variants, methods used to define related individuals, and methods used for genotype imputation using the 1000 Genomes (1000G) reference panel in MVP were previously described [19, 20]. Given large numbers of participant genotypes requiring calling and quality control, serial releases of genetic data were made available over time, in March 2017, August 2018, and September 2020. The **Supplemental Methods of** S2 File provide additional details.

### Genome-wide association study

The discovery GWAS cohort included MVP participants with genetic data released in the March 2017 genetic data freeze. The replication cohort comprised of separate MVP participants included in the August 2018 genetic data freeze who were not present in the March 2017 data freeze. Participants missing data on any LS7 component were excluded from analysis, leaving 142,404 in the discovery GWAS cohort and 45,766 in the replication cohort (**S1 Table of** S1 File). The majority of excluded participants had missing survey data, which was required for the behavioral components.

Genetic association analyses of IHS and BIH with SNP dosage (imputed from the 1000G reference panel) were examined within each race or ethnicity (European American = EUR, African American = AFR, Hispanic or Latino = HIS), and sex stratum. As a secondary analysis, we examined genetic association of SNP dosage with clinical IHS, behavioral IHS, clinical BIH and behavioral BIH. The harmonized ancestry and race/ethnicity (HARE) [21] method was used to define race or ethnicity. GWAS was performed on 29 million measured and imputed variants through linear regression for IHS, clinical IHS, and behavioral IHS and logistic regression for BIH, clinical BIH, and behavioral BIH, assuming an additive genomic model in PLINK 2.0 [22]. All models were adjusted for age and the first 10 ethnicity-specific principal components. Variants with imputation quality (R^2^) < 0.3 and estimated minor allele count ≤ 6 were excluded. We subsequently performed an inverse variance weighted multi-population meta-analysis (combined N = 142,404) for each of the ideal health traits using the Genome-Wide Association Meta-Analysis (GWAMA) software [23].

To investigate potential secondary signals, we performed conditional analysis adjusting for the top SNP in each genetic locus (defined as +/- 500KB of the top SNP) that reached genome-wide significance (p<5×10^−8^) in the IHS multi-population meta-analysis. Using these same top SNPs, we looked to replicate these associations in the replication cohort and meta-analyzed the combined discovery GWAS + replication cohort results (N = 188,170). We conducted sensitivity analysis for the top independent, significant SNPs removing 43,663 individuals with coronary artery disease (CAD), heart failure (HF) or ischemic stroke (IS) at enrollment.

### SNP annotations

Significantly associated SNPs were queried for known association with IHS components using the University of California Santa Cruz (UCSC) Genome Browser (https://genome.ucsc.edu/), the National Heart, Lung, and Blood Institute (NHLBI) Genome-Wide Repository of Associations Between SNPs and Phenotypes (GRASP) Catalog [24], GWAS Catalog [25], and the UKBiobank ICD PheWeb analysis of 1,403 ICD-based traits using SAIGE [http://pheweb.sph.umich.edu/SAIGE-UKB/]. In addition, LocusZoom [26] interactive plots of published GWAS results were used to examine known associations with IHS components using GWAS results. Additional details are provided in the **Supplemental Methods of** S2 File. We also examined lists of significant SNPs from the largest published GWAS on BMI [27], cholesterol [19], and blood pressure [14] for known associations. Linkage disequilibrium (LD) was calculated between significant IHS SNPs and nearby SNPs with known association with IHS components using Single Nucleotide Polymorphisms Annotator (SNiPA) [28].

### Phenome-wide association study

In order to avoid bias and overfitting, we performed PheWAS analyses in individuals from the September 2020 genetic data freeze who were not included in the discovery GWAS (**S1 Table of** S1 File). An ethnicity-specific weighted polygenic risk score of IHS (PRS_IHS_) was created based on 17 independent and genome-wide significant SNPs identified in the multi-population IHS meta-analysis and weights from the respective ethnicity-specific GWAS. The PRS_IHS_ was normalized by multiplying the PRS_IHS_ by the number of SNPs/sum of betas for 17 SNPs to allow for the effect size to be interpreted as a per-SNP effect in the PheWAS.

There were 21,209,658 prevalent ICD-9 diagnosis codes that were collapsed into ~1,800 binary phecodes [29]. Phecodes (N = 882) with at least 200 cases and 200 controls in 316,013 EUR, 99,325 AFR, and 40,688 HIS participants from the non-discovery GWAS cohort were used for subsequent Phenome-wide Association Study (PheWAS) analysis. The association between PRS_IHS_ and each phecode was tested using logistic regression models adjusting for age, sex, and the first 10 ethnicity-specific principal components with the ‘PheWAS’ R package [30] in R v3.2.0 [31]. PheWAS results were combined in a multi-population meta-analysis and the Bonferroni-corrected p-value threshold for significance was p < 5.67×10^−5^ (0.05/882). We also tested the association between each of the 17 individual SNPs and each phecode using logistic regression models adjusting for age, sex, and the first 10 ethnicity-specific principal components.

The PRS_IHS_ was created in the UK Biobank (UKB) including a subset of EUR individuals with less than third-degree relatedness and high confidence genotyping for all 17 SNPs of interest. The EUR-specific weights from the MVP IHS GWAS were used to create the PRS_IHS_ in UKB. We used phecodes (N = 1,084) derived from ICD-9 and ICD-10 diagnosis codes with at least 200 cases and 200 controls in 310,415 EUR from UKB for the PheWAS analysis to test the association between PRS_IHS_ and each phecode with logistic regression models, adjusting for age, sex and the first 10 principal components using the ‘PheWAS’ R package [30] in R v3.2.0 [31]. Phenotypes were coded as NA for sex-specific phenotypes (i.e., prostate cancer in a female).

### Genetic association of ideal cardiovascular health with mortality and cardiovascular disease outcomes

We obtained data for prevalent myocardial infarction (MI), IS, HF, and CAD, using ICD9/10 codes in the most recent freeze of MVP clinical data as of September 8, 2018. CAD was defined by prior occurrence of MI, revascularization, or chronic ischemic heart disease. We used ICD-10 codes to extract information on mortality outcomes from the latest freeze of National Death Index (NDI) data as of December 31, 2016, including all-cause mortality, CVD mortality, atherosclerotic CVD (ASCVD) mortality, and CAD mortality. Any analysis using mortality outcomes excluded individuals who were recruited after December 31, 2016. For the binary PRS_IHS_ variable (BPRS_IHS_), cases included individuals in the top 10th percentile of the PRS_IHS_ score and controls were everyone else.

We used the PRSice Polygenic Risk Score software [32] to select a best-fit PRS p-value threshold. We performed LD clumping in the discovery GWAS cohort and PRS tuning to find the best p-value threshold in the replication cohort. The default PRSice LD clumping (LD R^2^ < 0.1) & window-size (250 kb) was used. Our best-fit PRS_PRSice_ (p-value threshold < 0.49) was computed using 198,549 SNPs and their ancestry-specific betas. All PRS effect estimates were standardized and are to be interpreted as per-SD effects.

The association between PRS_PRSice_, PRS_IHS_, BPRS_IHS_, IHS, or BIH and each mortality/disease outcome was assessed using logistic regression models controlling for age, sex, race, and the first 10 ethnicity-specific principal components. We examined the effects of PRS_PRSice_, PRS_IHS_, BPRS_IHS_, IHS, and BIH on time to all-cause mortality using the Cox Proportional Hazards Model adjusted for age, sex, race, and the first 10 ethnicity-specific principal components. The proportional hazards assumption for the Cox regression model fit was tested using the ‘survival’ package [33, 34] in R. To investigate the effects of PRS_PRSice_, PRS_IHS_, BPRS_IHS_, IHS, and BIH on time to CVD death, CAD death and ASCVD death, we fit a cause-specific Cox proportional hazard regression model in competing risk using the ‘riskRegression’ package [35] in R, adjusting for age, sex, race, and the first 10 ethnicity-specific principal components. Participants in the discovery GWAS cohort and replication GWAS cohort (PRS tuning cohort) were excluded in the logistic and Cox regression analyses using the PRS. Primary analyses using IHS and BIH excluded participants in the discovery GWAS cohort. Secondary analyses were performed using the discovery GWAS cohort.

### Mendelian randomization

PRS_IHS_ was used as the genetic instrument in the two-sample Mendelian Randomization (MR) framework to examine for evidence of a causal association of ideal health with lower odds of CAD, IS, and HF. We extracted the effect size and standard error for the 17 independent, significant SNPs associated with IHS from external consortia: HF from the Heart Failure Molecular Epidemiology for Therapeutic Targets (HERMES) Consortium [36], IS from MEGASTROKE [37], and CAD from UK Biobank + Coronary Artery Disease Genome-wide Replication and Meta-analysis plus the Coronary Artery Disease (CARDIoGRAMplusC4D) consortium [38]. We used Egger’s regression for MR and inverse variance weighted regression using the ‘TwoSampleMR’ package [39, 40] and ‘MR-PRESSO’ package [41] in R.

## Results

### Sample characteristics

The discovery GWAS cohort included 142,404 participants: 119,129 EUR (83.7%), 16,495 AFR (11.6%), and 6,780 HIS (4.7%) (Table 1). The replication cohort included 45,766 participants with the majority EUR (82.3%), and the non-discovery non-replication GWAS cohort included 240,106 participants with 64.6% EUR. The proportion of AFR was higher in the non-discovery non-replication GWAS cohort (25.9%). The discovery GWAS, replication, and non-discovery non-replication GWAS cohorts had similar proportions of males—92.7%, 91.3%, and 90.9%, respectively. The participants’ age at enrollment in the discovery GWAS and replication cohorts were similar (mean discovery: 65.8 (11.2) years, replication: 65.9 (12.0) years), and the mean age at enrollment in the non-discovery non-replication GWAS cohort was somewhat younger (59.1 (14.7) years).

**Table 1:** Sample characteristics for discovery, replication, and non-discovery non-replication GWAS cohorts.

Mean (Standard deviation)		Discovery N = 142,404	Replication N = 45,766	Non-Discovery non-Replication N = 240,106
**Age, years**		65.8 (11.2)	65.9 (12.0)	59.1 (14.7)
**Male (n, %)**		131,951 (92.7%)	41,778 (91.3%)	218,376 (90.9%)
**Race/ethnicity**	**European American**	119,129 (83.7%)	37,646 (82.3%)	155,198 (64.6%)
	**African American**	16,495 (11.6%)	5,366 (11.7%)	62,078 (25.9%)
	**Hispanic American**	6,780 (4.7%)	2,754 (6.0%)	22,830 (9.5%)
**Body Mass Index, kg/m** ^**2**^		29.3 (5.5)	29.3 (5.6)	29.9 (6.0)
**Total Cholesterol, mg/dL**		169.6 (39.0)	170.5 (40.4)	175.6 (42.3)
**Plasma Glucose, mg/dL**		114.3 (41.3)	114.7 (43.5)	116.9 (50.7)
**Systolic Blood Pressure, mmHg**		130.9 (16.5)	131.8 (17.0)	130.9 (17.1)
Current Smoking* **(n, %)**		25,858 (18.2%)	8,179 (17.9%)	
Ideal Physical Activity* **(n, %)**		5,500 (3.9%)	1,812 (4.0%)	
**Binary Ideal Health (IHS** ≥ 9)* **(n, %)**		6,018 (4.2%)	1,697 (3.7%)	
**Ideal Health Score** * **, 0–12**		5.4 (1.8)	5.2 (1.8)	

*These values required questionnaire data; therefore, they are only available in the Discovery and Replication GWAS cohorts.

GWAS = genome-wide association study.

In the discovery GWAS cohort, 6,018 participants (4.2%) attained BIH with a mean (SD) IHS of 5.4 (1.8). The mean (SD) clinical and behavioral IHS were 3.2 (1.2) and 2.2 (1.1), respectively. In the replication cohort, BIH was present in 1,697 (3.7%) with a mean (SD) IHS of 5.2 (1.8). BMI, total cholesterol, plasma glucose, blood pressure, cigarette smoking, and physical activity level were similar in the discovery GWAS and replication cohorts (Table 1).

Among the 7 components of ideal CVH, plasma glucose was the health factor for which the greatest proportion of participants achieved ideal levels (N = 53,830, 37.8%). The majority of participants (N = 105,404, 74%) were considered to be in the poor category for physical activity (**S3 Table of** S1 File). **S4 Table of** S1 File includes sample characteristics of the discovery cohort stratified by race and sex. On average, men were older than women in the discovery cohort. A greater proportion of women achieved BIH compared to men. Hispanic women had the greatest proportion of individuals in the BIH category (14.6%).

### Genome-wide association study analysis

**Fig 1:**
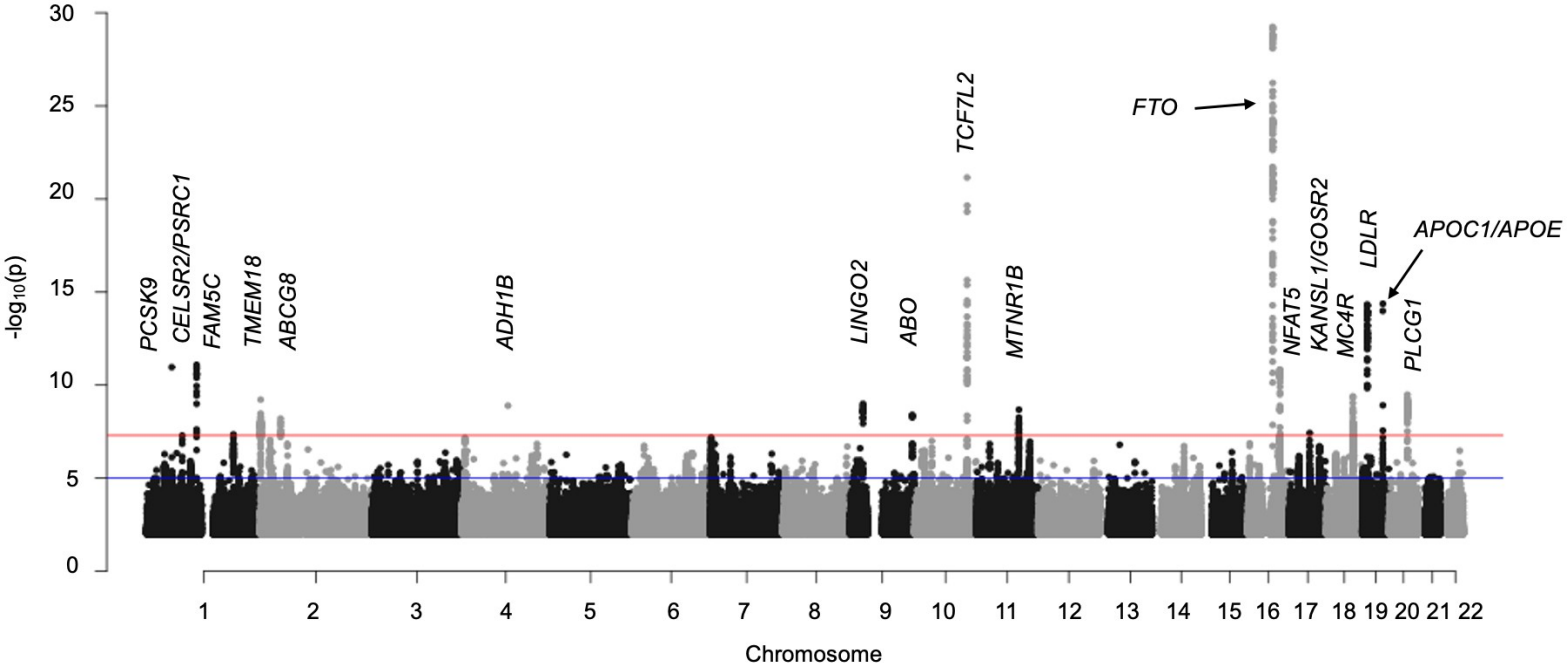
Multi-population ideal health score discovery GWAS Manhattan plot (N = 142,404). The negative log of the SNP p-value is plotted by chromosomal position (hg19) across the genome. Horizontal lines indicating genome-wide significant (p<5×10^−8^) and suggestive levels (p<1×10^−5^) are indicated by red and blue lines respectively. Transition between black and gray is used to define chromosome boundaries. Loci achieving genome-wide significance are annotated with the name of gene(s) in the region.

In the multi-population meta-analysis, 17 independent genome-wide significant (P<5×10^−8^) SNPs were associated with IHS (Fig 1, Table 2, **S1 Fig of** S2 File). The largest effect size was noted for the *PCSK9* locus, for which the T allele (rs11591147, effect allele frequency (EAF) = 0.01, P = 1.09×10^−11^) increased IHS by 0.20. In the EUR-only GWAS analysis, we identified 13 genome-wide significant loci associated with IHS. Although the direction of effect was the same across all race/ethnic groups, the 17 loci identified in the multi-population meta-analysis did not attain genome-wide significance in the AFR-only or HIS-only analyses (**S5 Table of** S1 File, **S2-S4 Figs of** S2 File). There was a suggestive association of the *APOC1/APOE* locus with IHS in the AFR-only cohort (P = 8.36×10^−6^). Among the 17 top SNPs from the IHS multi-population GWAS, 13 were associated with BIH at the Bonferroni-corrected level but not at the level of genome-wide significance (Table 2). We checked for secondary signals in each of the 17 loci by adjusting for the top SNP in each region and found no additional independently associated SNPs at these loci that reached genome-wide significance.

**Table 2:** Multi-population meta-analysis results for ideal health score and results for top 17 SNPs from binary ideal health genome-wide association study (N = 142,404).

Gene Region	CHR:POS	SNP	EA/OA	EAF	Ideal Health Score		Binary Ideal Health	
					BETA	P	OR	P
*CELSR2/PSRC1*	1:109817590	rs12740374	T/G	0.22	0.06	8.21E-12	1.07	**1.39E-03**
*FAM5C*	1:190306342	rs1171381	G/A	0.58	0.04	4.48E-08	1.00	8.98E-01
*PCSK9*	1:55505647	rs11591147	T/G	0.01	0.20	1.09E-11	1.30	**1.74E-04**
*ABCG8*	2:44096402	rs6544717	A/G	0.76	0.05	6.31E-09	1.13	**4.01E-07**
*TMEM18*	2:638838	rs180738835	D/I	0.78	0.05	6.05E-10	1.10	**2.32E-05**
*ADH1B*	4:100239319	rs1229984	T/C	0.96	0.11	1.27E-09	1.16	**2.31E-03**
*ABO*	9:136151806	rs600038	T/C	0.21	0.05	4.19E-09	1.07	4.96E-03
*LINGO2*	9:28415512	rs10968577	C/T	0.3	0.05	1.00E-09	1.07	**1.84E-03**
*TCF7L2*	10:114758349	rs7903146	C/T	0.29	0.07	7.01E-22	1.05	1.23E-02
*MTNR1B*	11:92672021	rs7112766	G/T	0.37	0.04	2.08E-09	1.07	**8.74E-04**
*FTO*	16:53800954	rs1421085	T/C	0.37	0.08	5.67E-30	1.08	**1.39E-04**
*NFAT5*	16:69663519	rs244417	C/T	0.39	0.05	1.47E-11	1.10	**1.61E-06**
*KANSL1/GOSR2*	17:44574284	rs371758411	D/I	0.26	0.05	3.75E-08	1.09	**2.52E-04**
*MC4R*	18:57811982	rs17700144	G/A	0.18	0.06	4.01E-10	1.09	**1.36E-03**
*LDLR*	19:11191197	rs114846969	A/G	0.12	0.08	4.67E-15	1.13	**2.51E-05**
*APOC1/APOE*	19:45413233	rs1065853	T/G	0.08	0.10	4.26E-15	1.18	**6.35E-07**
*PLCG1*	20:39760448	rs6029552	C/G	0.52	0.04	3.28E-10	1.06	2.98E-03

Nominally significant at Bonferroni-corrected threshold 0.05/17 = 2.94E-03.

EA = effect allele; CHR:POS = chromosome and position (hg19); OA = other allele; EAF = effect allele frequency; P = p-value; OR = odds ratio.

In the replication cohort, ten loci associated with IHS were significantly associated at a nominal P<0.05 level, and seven loci at a conservative Bonferroni-corrected threshold of P≤2.94×10^−3^. In the combined discovery GWAS cohort and replication cohort meta-analyses (N = 188,170), the effect direction was consistent and p-values for all SNPs remained genome-wide significant (Table 3). In a sensitivity analysis in the discovery GWAS cohort removing individuals with CVD at baseline (N = 98,741), beta coefficients and direction of effect for the 17 SNPs were similar to those from the overall discovery GWAS cohort (**S6 Table of** S1 File).

**Table 3:** Discovery-replication results for top 17 SNPs from the ideal health score multi-population genome-wide association study.

CHR	SNP	EA/OA	EAF	Discovery (N = 142,404)		Replication (N = 45,766)		Discovery + Replication (N = 188,170)	
				BETA	P	BETA	P	BETA	P
1	rs12740374	T/G	0.22	0.06	8.21E-12	0.07	7.42E-05*	0.06	**3.11E-15**
1	rs1171381	G/A	0.58	0.04	4.48E-08	0.03	3.96E-02	0.04	**5.90E-09**
1	rs11591147	T/G	0.01	0.20	1.09E-11	0.25	9.17E-06*	0.21	**7.09E-16**
2	rs6544717	A/G	0.76	0.05	6.31E-09	0.05	5.14E-03	0.05	**1.11E-10**
2	rs180738835	D/I	0.78	0.05	6.05E-10	0.02	3.414E-01	0.05	2.39E-09
4	rs1229984	T/C	0.96	0.11	1.27E-09	0.05	2.21E-01	0.10	2.19E-09
9	rs600038	T/C	0.21	0.05	4.19E-09	0.03	1.14E-01	0.05	**2.50E-09**
9	rs10968577	C/T	0.30	0.05	1.00E-09	0.01	3.88E-01	0.04	4.74E-09
10	rs7903146	C/T	0.29	0.07	7.01E-22	0.07	4.38E-06*	0.07	**1.58E-26**
11	rs7112766	G/T	0.37	0.04	2.08E-09	0.01	3.78E-01	0.04	8.63E-09
16	rs1421085	T/C	0.37	0.08	5.67E-30	0.08	2.26E-08*	0.08	**7.73E-37**
16	rs244417	C/T	0.39	0.05	1.47E-11	0.07	4.16E-06*	0.05	**5.23E-16**
17	rs371758411	D/I	0.26	0.05	3.75E-08	0.05	1.08E-02	0.05	**1.29E-09**
18	rs17700144	G/A	0.18	0.06	4.01E-10	0.02	2.35E-01	0.05	9.20E-10
19	rs114846969	A/G	0.12	0.08	4.67E-15	0.07	1.29E-03*	0.08	**2.79E-17**
19	rs1065853	T/G	0.08	0.10	4.26E-15	0.14	1.40E-08*	0.10	**1.24E-21**
20	rs6029552	C/G	0.52	0.04	3.28E-10	0.02	7.76E-02	0.04	**1.40E-10**

*Significant at Bonferroni-corrected threshold 0.05/17 = 2.94E-03.

Bolded text indicates that combined discovery + replication p-value is smaller than discovery p-value.

CHR = chromosome; EA = effect allele; OA = other allele; EAF = effect allele frequency; P = p-value.

In the BIH GWAS, 14 genetic loci were associated at a genome-wide significance level in the discovery cohort; however, the majority of top SNPs in these regions had small minor allele frequencies, most less than 0.01, and very large odds ratios (OR) (**S7 Table of** S1 File). Further external replication is needed to confirm the validity of these loci.

We performed secondary GWAS analyses of clinical and behavioral ideal CVH. In the GWAS for clinical IHS, 49 genetic loci were associated at the genome-wide significance level, and the two genetic variants most significantly associated were located near *TCF7L2* (P = 7.43×10^−87^) and *APOE/APOC1* (P = 2.05×10^−65^) (**S8 Table of** S1 File). In the GWAS analysis for clinical BIH, 16 genetic loci were significantly associated in the multi-population meta-analysis (**S9 Table of** S1 File). 18 loci were associated with behavioral IHS and 4 loci associated with behavioral BIH (**S10 and S11 Tables of** S1 File). Of the 17 loci significantly associated with overall IHS, 15 were associated with at least one of the clinical or behavioral ideal CVH measures. Full GWAS summary statistics can be found in dbGaP (https://www.ncbi.nlm.nih.gov/gap/) under the MVP accession (phs001672).

### Prior SNP associations with multiple ideal health traits

Each of the top 17 SNPs in the MVP IHS GWAS were associated with a wide set of cardiometabolic diseases and related risk factors in the literature (**S2 Table of** S1 File). In numerous prior GWAS studies, the identified SNPs also have known or suggestive protective pleiotropic associations with the ideal health components. For the clinical components, 14 loci were associated with cholesterol, 10 with blood pressure, and 10 with glucose or T2D. Additionally, 15 loci have known associations with BMI. *LDLR*, *PCSK9*, *ABO*, and *ABCG8* loci have known association with LDL cholesterol and physical activity interaction [42]. *TMEM18*, *LINGO2*, and *FAM5C* have been associated with BMI in physically active individuals [43]. *ABCG8* is associated with smoking initiation (ever regular vs never regular), and *KANSL1/GOSR2* is associated with age of smoking initiation [44]. *FTO* and *LINGO2* have suggestive associations with nicotine dependence [45, 46]. The alleles found to be associated with better IHS for these SNPs are the previously reported protective alleles for diseases and risk factors. Additionally, the lead SNP in many of the implicated loci, such as *TCF7L2*, *CELSR2/PSRC1*, and *PCSK9*, were associated with multiple cardiovascular risk factors and outcomes in the UKBiobank ICD PheWeb (**S12 Table of** S1 File).

### Genetic instrument characteristics

With the 17 independent SNPs from the IHS multi-population meta-analysis, we created an ethnicity-specific weighted PRS_IHS_ for EUR, AFR, and HIS participants. This PRS_IHS_ was significantly associated with IHS among all groups. The F statistics for the association between ideal health score and the instrument (PRS of ideal health score) were 1158 in EUR, 148.9 in AFR, and 96.7 in HIS, which suggests low risk for weak instrument bias. The heritability of IHS was 0.125 for EUR, and the EUR PRS_IHS_ explained 0.7% of IHS variance in those participants. The proportion of variance explained by the PRS_IHS_ was 0.6% for AFR and 0.8% for HIS. The best-fit PRS_PRSice_ with 198,549 SNPs (p-value threshold < 0.49) selected using PRSice explained roughly 1% (or 0.009) of variation in IHS in the replication cohort.

### Phenome-wide association study

**Fig 2:**
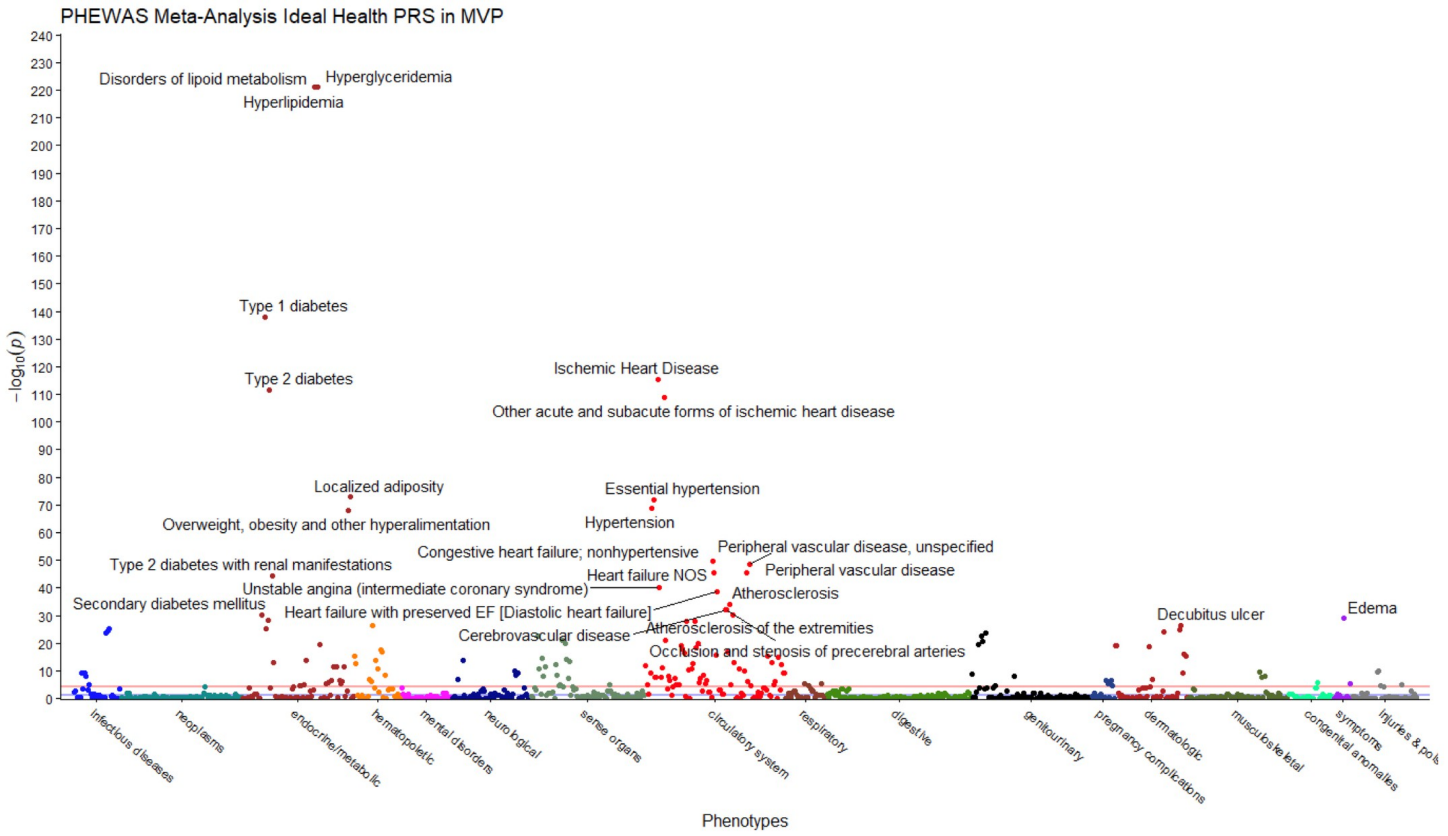
Multi-population PheWAS Manhattan plot: Ideal health score polygenic risk score vs disease phecode. The negative log of the p-value is plotted for each of 882 disease phenotypes or “phecodes” with at least 200 cases and 200 controls in MVP. The horizontal red line indicates the statistically significant threshold (P < 5.67×10^−5^). Each color represents a disease category as defined on the x-axis. Loci achieving p<1×10^−30^ are annotated with the phecode description.

There were 163 phecodes in the multi-population meta-analysis, 148 phecodes in EUR, 26 phecodes in AFR, and 20 phecodes in HIS significantly associated with PRS_IHS_ at the Bonferroni-corrected p-value (P < 5.67×10^−5^, Fig 2). In the multi-population meta-analysis, protective associations were noted for numerous phecodes denoting clinically apparent CVD, including ischemic heart disease (OR = 0.96, P = 4.64×10^−116^), atherosclerosis (OR = 0.95, P = 5.85×10^−35^), congestive HF (OR = 0.96, P = 1.65×10^−50^), peripheral vascular disease (OR = 0.96, P = 2.26×10^−46^), cerebrovascular disease (OR = 0.97, P = 8.60×10^−33^), aortic valve disease (OR = 0.98, P = 6.81 ×10^−10^), cardiac arrest and ventricular fibrillation (OR = 0.98, P = 3.08×10^−9^), pulmonary heart disease (OR = 0.98, P = 6.02×10^−9^), and atrial fibrillation and flutter (OR = 0.98, P = 5.70×10^−8^) (**S13 Table of** S1 File). Significantly lower odds of CVD risk factors related to IHS components (T2D, hypertension, and obesity) were also significantly noted in the PheWAS. The top phecodes associated with PRS_IHS_ were hyperlipidemia, disorders of lipid metabolism, and hyperglyceridemia (P < 5×10^−324^).

In AFR only and HIS only MVP PheWAS, PRS_IHS_ was significantly associated with ischemic heart disease, HF, hyperlipidemia and T2D **(S14 and S15 Tables of** S1 File). Additionally, PRS_IHS_ was significantly associated peripheral vascular disease and cerebrovascular disease in the AFR only MVP PheWAS **(S14 Table of** S1 File).

Results from the UKB PheWAS of PRS_IHS_ were consistent with MVP results. There were 40 phecodes significantly associated with PRS_IHS_ at the Bonferroni-corrected p-value (P < 4.61×10^−5^, **S13 Table of** S1 File) in UKB. PRS_IHS_ was significantly associated with ischemic heart disease (OR = 0.96, P = 6.57×10^−46^), coronary atherosclerosis (OR = 0.95, P = 9.87×10^−43^), MI (OR = 0.95, P = 5.40×10^−31^), HF (OR = 0.95, P = 2.54×10^−14^), cardiac congenital anomalies (OR = 0.95, P = 6.73×10^−12^), peripheral vascular disease (OR = 0.96, P = 1.76×10^−9^), atrial fibrillation (OR = 0.98, P = 1.08 ×10^−5^), hypertension (OR = 0.98, P = 1.82×10^−25^), hyperlipidemia (OR = 0.94, P = 3.81×10^−131^), type 2 diabetes (OR = 0.94, P = 2.19×10^−73^), and obesity (OR = 0.97, P = 1.10×10^−13^) **(S13 Table of** S1 File).

When we examined the PheWAS for each of the individual 17 SNPs in MVP, findings were consistent with protective associations both for the expected risk factor(s) and cardiometabolic outcomes. In the multi-population analyses, 10 of the 17 SNPs (*CELSR2/PSRC1*, *PCSK9*, *ABCG8*, *ABO*, *TCF7L2*, *FTO*, *NFAT5*, *KANSL2/GOSR2*, *APOC1/APOE*, and *LDLR*) were protective against ischemic heart disease. *CELSR2/PSRC1*, *TMEM18*, *ADH1B*, *ABO*, *FTO*, *NFAT5*, *KANSL2/GOSR2*, and *LDLR* were protective against HF. *CELSR2/PSRC1*, *ABO*, *TCF7L2*, *MTNR1B*, and *LDLR* were protective against cerebrovascular disease. In addition, *CELSR2/PSRC1*, *PCSK9*, *ABCG8*, *ABO*, *TCF7L2*, *FTO*, *NFAT5*, *APOC1/APOE*, *LDLR*, and *PLCG1* were found to be protective against hyperlipidemia **(S16 Table of** S1 File).

### Association of ideal cardiovascular health with mortality and cardiovascular disease outcomes

In the non-discovery GWAS cohort, IHS was significantly associated with reduced risk of CVD outcomes (MI, HF, IS, and CAD), death from all-causes, as well as death from CAD, CVD, and ASCVD. BIH was associated with reduced risk of MI (OR = 0.34, P = 4.32×10^−10^), HF (OR = 0.40, P = 3.52×10^−14^), CAD (OR = 0.37, P < 2×10^−16^), and IS (OR = 0.53, P = 3.59×10^−5^), as well as reduced death from ASCVD (OR = 0.35, P = 0.04) (Table 4). In the discovery GWAS cohort, BIH was also associated with reduced risk of death from all causes (OR = 0.69, P = 4.34×10^−10^), death from CAD (OR = 0.51, P = 3.97×10^−5^), and death from CVD (OR = 0.55, P = 2.98×10^−7^). In the non-discovery and non-replication GWAS cohort, logistic regression analysis showed a protective association of genetically-defined IHS (PRS_IHS_ and PRS_PRSice_) on CVD death, CAD deaths, ASCVD deaths, all deaths, MI, IS, CAD and HF. BPRS_IHS_ (top 10^th^ percentile of PRS vs everyone else) was associated with reduced odds of MI (OR = 0.80, P = 3.38×10^−11^), HF (OR = 0.88, P = 2.48×10^−6^), and CAD (OR = 0.84, P < 2×10^−16^) (**S17 Table of** S1 File).

**Table 4:** Logistic regression analysis of cardiovascular disease outcomes and mortality outcomes using ideal cardiovascular health or genetically-defined ideal cardiovascular health.

**Outcome**	**N Cases**	**N Controls**	**PRS**_**PRSice**_ **(198,549 SNPs)**			**PRS**_**IHS**_ **(17 SNPs)**		**B PRS**		
			**OR**	**P**		**OR**	**P**	**OR**		**P**
Myocardial Infarction	16,418	223,688	0.85	**<2E-16**		0.90	**< 2E-16**	0.80		**3.38E-11**
Heart Failure	28,266	211,840	0.86	**<2E-16**		0.92	**< 2E-16**	0.88		**2.48E-06**
Ischemic Stroke	15,951	224,155	0.91	**<2E-16**		0.96	**1.10E-05**	0.97		0.304
CAD (MI, CIHD or Revas)	60,661	179,445	0.86	**<2E-16**		0.91	**< 2E-16**	0.84		**< 2E-16**
All deaths	16,719	223,387	0.93	**<2E-16**		0.96	**3.06E-06**	0.94		0.071
CAD deaths	2,608	237,498	0.87	**7.09E-13**		0.90	**3.35E-06**	0.88		0.094
CVD deaths	4,625	235,481	0.89	**2.98E-15**		0.92	**2.33E-06**	0.98		0.754
ASCVD deaths	3,206	236,900	0.88	**7.54E-13**		0.90	**1.36E-06**	0.94		0.418
**Outcome**	**N Cases**	**N Controls**	**Ideal Health Score**				**Binary Ideal Health** (IHS ≥ 9)			
			**OR**		**P**		**OR**		**P**	
Myocardial Infarction	2879	42,887	0.78		**< 2E-16**		0.34		**4.32E-10**	
Heart Failure	4994	40,772	0.77		**< 2E-16**		0.40		**3.52E-14**	
Ischemic Stroke	2382	43,384	0.88		**< 2E-16**		0.53		**3.59E-05**	
CAD (MI, CIHD or Revas)	15,335	30,431	0.80		**< 2E-16**		0.37		**< 2E-16**	
All deaths	1612	32,373	0.97		**0.03**		0.78		0.11	
CAD deaths	230	33,755	0.84		**1.8E-05**		0.32		0.05	
CVD deaths	434	33,551	0.89		**3.68E-05**		0.56		0.07	
ASCVD deaths	280	33,705	0.86		**3.49E-05**		0.35		**0.04**	

Significant at 0.05 significance level.

PRS = polygenic risk score; BPRS = binary PRS (top 10^th^ percentile of PRS vs everyone else); OR = odds ratio; P = p-value; CAD = coronary artery disease (MI, revascularization, or chronic ischemic heart disease); Revas = revascularization; CIHD = chronic ischemic heart disease; CVD = cardiovascular disease, ASCVD = atherosclerotic cardiovascular disease.

In Cox regression analysis, PRS_IHS_, PRS_PRSice_ and IHS were significantly associated with decreased risk of total mortality. In the competing risk model, PRS_IHS_, PRS_PRSice_, and IHS were significantly associated with reduced risk of CVD death, CAD deaths, and ASCVD deaths. In the non-discovery GWAS cohort, BIH was associated with reduced risk of ASCVD deaths (HR = 0.35, P = 0.04) (Table 5). Furthermore, in the GWAS cohort, BIH was also associated with reduced risk of total mortality (HR = 0.71, P = 7.20×10^−10^), CAD death (HR = 0.51, P = 3.32×10^−5^), CVD death (HR = 0.55, P = 2.48×10^−7^), and ASCVD deaths (HR = 0.54, P = 2.28×10^−5^) (**S18 Table of** S1 File).

**Table 5:** Cox regression and competing risk analysis of mortality outcomes.

Trait	Hazard Ratios	P-value	Deaths	N
***All Deaths***				
B PRS	0.95	0.09	16,719	240,106
PRS_IHS_ (17 SNPs)	0.96	**3.77E-6**	16,719	240,106
PRS_PRSice_ (198,549 SNPs)	0.93	**<2E-16**	16,719	240,106
Binary Ideal Health (IHS ≥ 9)	0.78	0.09	1,612	33,985
Ideal Health Score	0.97	**0.02**	1,612	33,985
***Coronary Artery Disease Deaths***				
B PRS	0.88	0.10	2,608	240,106
PRS_IHS_ (17 SNPs)	0.90	**3.09E-6**	2,608	240,106
PRS_PRSice_ (198,549 SNPs)	0.86	**2.63E-13**	2,608	240,106
Binary Ideal Health (IHS ≥ 9)	0.32	0.05	230	33,985
Ideal Health Score	0.84	**1.75E-05**	230	33,985
***Cardiovascular Disease Deaths***				
B PRS	0.98	0.78	4,625	240,106
PRS_IHS_ (17 SNPs)	0.92	**2.36E-6**	4,625	240,106
PRS_PRSice_ (198,549 SNPs)	0.89	**8.96E-16**	4,625	240,106
Binary Ideal Health (IHS ≥ 9)	0.56	0.07	434	33,985
Ideal Health Score	0.89	**3.49E-05**	434	33,985
***Atherosclerotic Cardiovascular Disease Deaths***				
B PRS	0.95	0.44	3,206	240,106
PRS_IHS_ (17 SNPs)	0.91	**1.27E-6**	3,206	240,106
PRS_PRSice_ (198,549 SNPs)	0.88	**2.55E-13**	3,206	240,106
Binary Ideal Health (IHS ≥ 9)	0.35	**0.04**	280	33,985
Ideal Health Score	0.86	**3.38E-05**	280	33,985

Significant at 0.05 significance level.

PRS = polygenic risk score; BPRS = binary PRS (top 10% of PRS vs everyone else).

### Mendelian randomization

**Fig 3:**
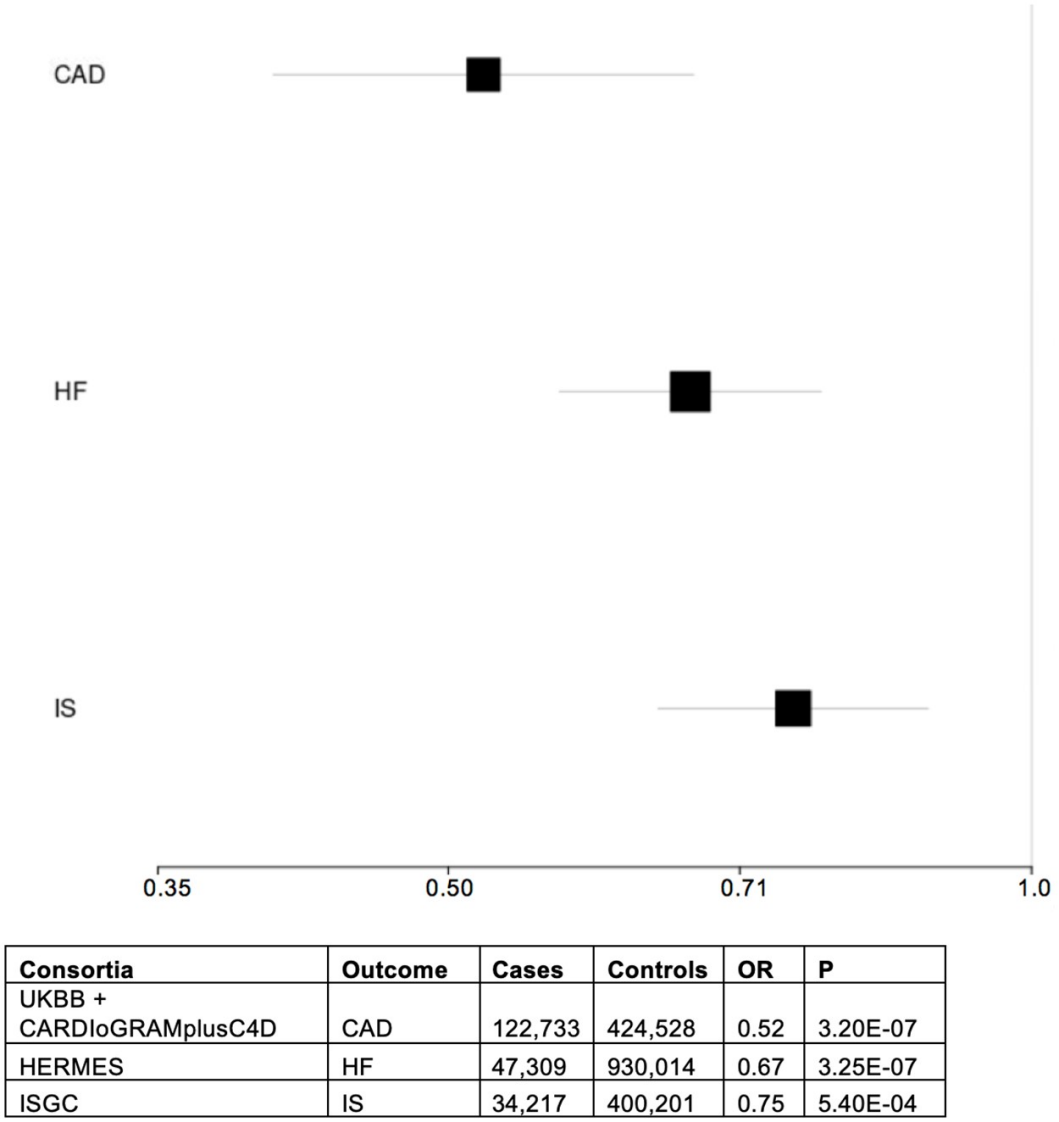
Two-sample Mendelian randomization results & forest plot. The forest plot shows the OR and 95% confidence interval for each CVD outcome (CAD, HF, and IS). The table at the bottom of the figure describes the external consortia used for each outcome, along with the numbers of cases, controls, OR, and p-values. CAD = coronary artery disease; HF = heart failure, IS = ischemic stroke; OR = odds ratio; P = p-value; UKBB = UK Biobank; CARDIoGRAMplusC4D = Coronary Artery Disease Genome-wide Replication and Meta-analysis plus the Coronary Artery Disease consortium; HERMES = Heart Failure Molecular Epidemiology for Therapeutic Targets Consortium; ISGC = International Stroke Genetics Consortium.

Based on inverse variance weighted regression for two-sample MR, genetically-defined IHS was associated with lower odds of CAD (OR = 0.52, P = 3.20×10^−7^), HF (OR = 0.67, P = 3.25×10^−7^), and IS (OR = 0.75, P = 5.4×10^−4^) (Fig 3). MR Egger regression, a more conservative approach, supported the association between genetically-defined IHS and CAD (OR = 0.40, P = 0.05). MR Egger intercepts were non-significant, suggesting no evidence of horizontal pleiotropy. Additional sensitivity analyses using MR-PRESSO’s outlier correction method gave similar results to the IVW regression and was significant for all outcomes (**S19 Table of** S1 File).

## Discussion

We discovered 17 novel genetic loci associated with IHS in the multi-population meta-analysis of EUR, AFR, and HIS participants of MVP. Our finding of a strong association of the *APOC1/APOE* region with clinical IHS is concordant with the prior reported association in a EUR population in the CHARGE consortium [16]. SNPs in all 17 loci have previously been associated in GWAS studies with multiple individual components of IHS, suggesting these particular loci are key nodes with simultaneous effects on several health factors and behaviors in a direction that is associated with survival free of CVD or its recurrence.

Genetically defined IHS is associated with lower all-cause mortality, CVD deaths, ASCVD deaths, and CAD deaths, as well as a broad spectrum of CVDs, known CVD risk factors and a range of other diseases. We extend prior studies in our finding that ideal CVH (excluding diet component) is strongly associated with lower CVD deaths, CAD deaths, ASCVD deaths, and multiple other CVD outcomes. Two-sample MR provided strong evidence for genetically influenced associations of ideal CVH with lower occurrence of CAD, HF, and IS. These results provided further support for consistency of associations, in a protective direction, of multiple health factors with maintenance of health. While observational evidence is consistent with lower risk of CVD from ideal CVH [2, 47], randomized control trials conducted decades ago did not show promising results in decreasing disease risk from modifying a subset of the risk factors related to ideal CVH [9–11]. While our current study was not designed to simulate the potential impact of multiple simultaneous preventive interventions, the finding of a protective genetic association for the overall PRS, as well as for each individual SNP in the PRS, shows a consistently protective direction of effect across all major health factors comprising IHS in the multiethnic MVP biobank cohort and consistency of associations in the UK Biobank that support current public health recommendations.

The genetic associations with IHS in the GWAS meta-analysis were consistent across race and ethnic groups, although the magnitude of effect varied. 13 of the 17 SNPs significant in the meta-analysis attained genome-wide significance in the EUR-only GWAS of IHS and the failure to attain genome-wide significance in AFR-only and HIS-only analyses is likely related to the limited sample size when compared to EUR. While these findings are consistent with the presence of protective associations across race or ethnic groups, further studies in larger cohorts are needed to define the role of individual genetic variants associated with ideal CVH in AFR and HIS populations.

In numerous prior GWAS studies and in unpublished UKBiobank browser results, the identified SNPs have known or suggestive associations with a wide set of cardiometabolic diseases and related risk factors as well as the ideal health components. The alleles found to be associated with better IHS are the previously reported protective alleles for diseases and risk factors, supporting the role of variation in these gene regions in CVD and related disorders.

In the MVP PheWAS, genetically defined ideal CVH was associated with lower odds of a broad spectrum of CVD outcomes and related cardiometabolic diseases, including congestive HF, peripheral vascular disease, cerebrovascular disease, and atrial fibrillation. Furthermore, PRS_IHS_ was associated with lower odds of CVD risk factors such as hyperlipidemia, hypercholesterolemia, T2D, and hypertension, as well as hypertensive chronic kidney disease, and morbid obesity. The PheWAS findings in the large cohort of UKB are largely consistent in associated outcomes and protective direction of effect for persons of European descent in MVP for the overall PRS_IHS_. In Cox regression survival analysis, PRS_IHS_ and IHS were associated with lower risk of death from all-causes as well as death from CAD, CVD, and ASCVD. The follow-up MR analysis provide strong evidence of an association in a protective direction of ideal CVH on CAD, HF and IS.

Our study is the largest to date to investigate the genetic basis of ideal CVH and the first to incorporate large numbers of participants from populations under-represented in genetic and health research, particularly those of AFR and HIS descent. These results were obtained from a single large study in the VA healthcare system, providing less heterogeneity compared to meta-analyses of multiple different cohorts; however, there are still several study limitations. First, we could not confirm fasting status for more than half of our participants and therefore, non-fasting plasma glucose values were potentially included. Second, we were unable to measure the impact of the diet component of Life’s Simple 7 due to the low prevalence of individuals with ideal diet in the VA. Third, we acknowledge that our power to detect associations may be limited by the use of categorical measures in the IHS, particularly given prior studies that demonstrate net benefit from reduction across a continuous range of measured levels of clinical and behavioral risk factors. Fourth, MVP participants were drawn from the VA healthcare system, and although our analyses included tens of thousands of women participants, the majority of our participants were older males, so we were limited in ability to robustly explore sex interactions; further, we have reported that the baseline balance of risk factors may differ somewhat between users of the VA and other contemporary populations [18]. However, we note substantial consistency between PheWAS results in MVP and in UKB, which includes similar proportions of women and men. Fifth, we acknowledge that categorized versus continuous measures of clinical and behavioral risk factors used to create a combined ideal health score measure may result in reduced power. Finally, while we note that genetic variants associated with IHS did have multiple protective associations with several phenotypes in the PheWAS, results of our conservative MR tests did not violate pleiotropy assumptions, although we cannot entirely exclude a role for unmeasured pleiotropy and residual confounding.

In conclusion, we identified 17 genetic loci associated with IHS, and these associations are consistent across all race/ethnic groups tested. Beyond the known association of the *APOE* locus, each of the other identified loci are known to be associated with several individual health factors, as well as the overall association with IHS, and the allelic direction of effect was as expected. From the available evidence, the IHS is strongly associated with lower risk of CHD, HF, and other CVDs. We confirm and extend known associations of IHS with favorable levels of a range of CVD outcomes and further support the potential beneficial effects of IHS across a broad range of CVD and other conditions in a multi-ethnic population. Genetically defined IHS predicts significantly lower risk of CVDs, all-cause mortality, and mortality from various CVD outcomes in survival and logistic regression analyses. MR analysis strengthens the evidence for the favorable association of ideal CVH on CVD outcomes. Our data lends further support from a large, comprehensive, multi-ethnic cohort for prevention guidelines that include interventions to modify multiple established CVD risk factors and consideration of genetic evaluation of IHS in future prevention trials in diverse populations to evaluate multiple risk factor interventions.

## Supporting information

S1 File (XLSX)

S2 File (DOCX)
