# Supplementary material for: Genome-wide and phenome-wide analysis of ideal cardiovascular health in the VA Million Veteran Program
Source: PLoS One. 2022 May 25;17(5):e0267900. doi: 10.1371/journal.pone.0267900 (PMC9132265; doi:10.1371/journal.pone.0267900)
Supplement: S2 File — (DOCX) [file pone.0267900.s002.docx]

**SUPPLEMENTAL MATERIALS**

**LIST OF VA MILLION VETERAN PROGRAM MEMBERS**

**MVP Executive Committee**

- Co-Chair: J. Michael Gaziano, M.D., M.P.H.
- Co-Chair: Sumitra Muralidhar, Ph.D.
- Rachel Ramoni, D.M.D., Sc.D., Chief VA Research and Development Officer
- Jean Beckham, Ph.D.
- Kyong-Mi Chang, M.D.
- Christopher J. O’Donnell, M.D., M.P.H.
- Philip S. Tsao, Ph.D.
- James Breeling, M.D., Ex-Officio
- JP Casas Romero, M.D., Ph.D., Ex-Officio

**MVP Program Office**

- Sumitra Muralidhar, Ph.D.
- Jennifer Moser, Ph.D.

**MVP Recruitment/Enrollment**

- Recruitment/Enrollment Director/Deputy Director, Boston – Stacey B. Whitbourne, Ph.D.; Jessica V. Brewer, M.P.H.
- MVP Coordinating Centers
  - Clinical Epidemiology Research Center (CERC), West Haven – Mihaela Aslan, Ph.D.
  - Cooperative Studies Program Clinical Research Pharmacy Coordinating Center, Albuquerque – Todd Connor, Pharm.D.; Dean P. Argyres, B.S., M.S.
  - Genomics Coordinating Center, Palo Alto – Philip S. Tsao, Ph.D.
  - MVP Boston Coordinating Center, Boston - J. Michael Gaziano, M.D., M.P.H.
  - MVP Information Center, Canandaigua – Brady Stephens, M.S.
- VA Central Biorepository, Boston – Mary T. Brophy M.D., M.P.H.; Donald E. Humphries, Ph.D. Luis E. Selva, Ph.D.
- MVP Informatics, Boston – Nhan Do, M.D.; Shahpoor Shayan
- MVP Data Operations/Analytics, Boston – Kelly Cho, Ph.D.

**MVP Science**

- Science Operations – Christopher J. O’Donnell, M.D., M.P.H
- Genomics Core - Christopher J. O’Donnell, M.D., M.P.H.; Saiju Pyarajan Ph.D.; Philip S. Tsao, Ph.D.
- Phenomics Core- Kelly Cho, M.P.H, Ph.D.
- Data and Computational Sciences – Saiju Pyarajan, Ph.D.
- Statistical Genetics – Elizabeth Hauser, Ph.D.; Yan Sun, Ph.D.; Hongyu Zhao, Ph.D.

**Current MVP Local Site Investigators**

- Atlanta VA Medical Center (Peter Wilson, M.D.)
- Bay Pines VA Healthcare System (Rachel McArdle, Ph.D.)
- Birmingham VA Medical Center (Louis Dellitalia, M.D.)
- Central Western Massachusetts Healthcare System (Kristin Mattocks, Ph.D., M.P.H.)
- Cincinnati VA Medical Center (John Harley, M.D., Ph.D.)
- Clement J. Zablocki VA Medical Center (Jeffrey Whittle, M.D., M.P.H.)
- VA Northeast Ohio Healthcare System (Frank Jacono, M.D.)
- Durham VA Medical Center (Jean Beckham, Ph.D.)
- Edith Nourse Rogers Memorial Veterans Hospital (John Wells., Ph.D.)
- Edward Hines, Jr. VA Medical Center (Salvador Gutierrez, M.D.)
- Veterans Health Care System of the Ozarks (Gretchen Gibson, D.D.S., M.P.H.)
- Fargo VA Health Care System (Kimberly Hammer, Ph.D.)
- VA Health Care Upstate New York (Laurence Kaminsky, Ph.D.)
- New Mexico VA Health Care System (Gerardo Villareal, M.D.)
- VA Boston Healthcare System (Scott Kinlay, M.B.B.S., Ph.D.)
- VA Western New York Healthcare System (Junzhe Xu, M.D.)
- Ralph H. Johnson VA Medical Center (Mark Hamner, M.D.)
- Columbia VA Health Care System (Roy Mathew, M.D.)
- VA North Texas Health Care System (Sujata Bhushan, M.D.)
- Hampton VA Medical Center (Pran Iruvanti, D.O., Ph.D.)
- Richmond VA Medical Center (Michael Godschalk, M.D.)
- Iowa City VA Health Care System (Zuhair Ballas, M.D.)
- Eastern Oklahoma VA Health Care System (Douglas Ivins, M.D.)
- James A. Haley Veterans’ Hospital (Stephen Mastorides, M.D.)
- James H. Quillen VA Medical Center (Jonathan Moorman, M.D., Ph.D.)
- John D. Dingell VA Medical Center (Saib Gappy, M.D.)
- Louisville VA Medical Center (Jon Klein, M.D., Ph.D.)
- Manchester VA Medical Center (Nora Ratcliffe, M.D.)
- Miami VA Health Care System (Hermes Florez, M.D., Ph.D.)
- Michael E. DeBakey VA Medical Center (Olaoluwa Okusaga, M.D.)
- Minneapolis VA Health Care System (Maureen Murdoch, M.D., M.P.H.)
- N. FL/S. GA Veterans Health System (Peruvemba Sriram, M.D.)
- Northport VA Medical Center (Shing Shing Yeh, Ph.D., M.D.)
- Overton Brooks VA Medical Center (Neeraj Tandon, M.D.)
- Philadelphia VA Medical Center (Darshana Jhala, M.D.)
- Phoenix VA Health Care System (Samuel Aguayo, M.D.)
- Portland VA Medical Center (David Cohen, M.D.)
- Providence VA Medical Center (Satish Sharma, M.D.)
- Richard Roudebush VA Medical Center (Suthat Liangpunsakul, M.D., M.P.H.)
- Salem VA Medical Center (Kris Ann Oursler, M.D.)
- San Francisco VA Health Care System (Mary Whooley, M.D.)
- South Texas Veterans Health Care System (Sunil Ahuja, M.D.)
- Southeast Louisiana Veterans Health Care System (Joseph Constans, Ph.D.)
- Southern Arizona VA Health Care System (Paul Meyer, M.D., Ph.D.)
- Sioux Falls VA Health Care System (Jennifer Greco, M.D.)
- St. Louis VA Health Care System (Michael Rauchman, M.D.)
- Syracuse VA Medical Center (Richard Servatius, Ph.D.)
- VA Eastern Kansas Health Care System (Melinda Gaddy, Ph.D.)
- VA Greater Los Angeles Health Care System (Agnes Wallbom, M.D., M.S.)
- VA Long Beach Healthcare System (Timothy Morgan, M.D.)
- VA Maine Healthcare System (Todd Stapley, D.O.)
- VA New York Harbor Healthcare System (Scott Sherman, M.D., M.P.H.)
- VA Pacific Islands Health Care System (George Ross, M.D.)
- VA Palo Alto Health Care System (Philip Tsao, Ph.D.)
- VA Pittsburgh Health Care System (Patrick Strollo, Jr., M.D.)
- VA Puget Sound Health Care System (Edward Boyko, M.D.)
- VA Salt Lake City Health Care System (Laurence Meyer, M.D., Ph.D.)
- VA San Diego Healthcare System (Samir Gupta, M.D., M.S.C.S.)
- VA Sierra Nevada Health Care System (Mostaqul Huq, Pharm.D., Ph.D.)
- VA Southern Nevada Healthcare System (Joseph Fayad, M.D.)
- VA Tennessee Valley Healthcare System (Adriana Hung, M.D., M.P.H.)
- Washington DC VA Medical Center (Jack Lichy, M.D., Ph.D.)
- W.G. (Bill) Hefner VA Medical Center (Robin Hurley, M.D.)
- White River Junction VA Medical Center (Brooks Robey, M.D.)
- William S. Middleton Memorial Veterans Hospital (Robert Striker, M.D., Ph.D.)

**SUPPLEMENTAL METHODS**

Genotype Data and Genetic Quality Control

Blood specimens were collected at recruitment sites across the country then shipped within 24 hours to the VA Central Biorepository in Boston, MA for processing and storage. Duplicate samples were excluded from analysis. Additional exclusion criteria included:

samples with observed heterozygosity greater than the expected heterozygosity, missing genotype call rate greater than 2.5%, and incongruence between sex inferred from genetic information and gender extracted from phenotype data. KING software [1] was used to measure relatedness between individuals in the sample. One individual was removed from each pair of related individuals. Where more than one pair of individuals from a family were present, we kept one individual and excluded the rest of the family members. These quality control measures follow standard quality control procedures for genetic studies.

EIGENSOFT software was used to conduct ethnicity-specific principal component analysis. This allows correction for potential confounding due to population stratification, or differences in allele frequency between subpopulations as a result of systematic ancestry differences, that can cause spurious associations in GWAS [2,3].

Genotype Imputation

Variants with genotype missingness greater than 5% or those with observed allele frequency different from their expected allele frequency based on the 1000 Genomes Project [4] reference data were excluded. Pre-phasing, or statistically estimating each individual’s haplotype to reduce computation costs of imputation [5], was performed using EAGLE v2 [6], and Minimac3 software [5] was used to impute the genotypes from the 1000 Genomes Project [4] phase 3, version 5 reference panel into MVP participants [2].

GWAS

Subsequently, we performed variant-level quality control using the ‘EasyQC’ R package [7] and the following exclusion criteria: imputation quality (RSq) <0.3 and estimated minor allele count (eMAC) ≤ 6. eMAC is calculated as the minor allele count multiplied by the imputation quality (RSq), therefore taking into account the imprecision of this technique.

We created chromosome-specific quantile-quantile (Q-Q) plots to examine the relationship between observed and expected p-values for SNPs under the null distribution. The genomic control inflation factor λ could be inflated as a result of population substructure, relatedness, and genotyping errors [8–10]. This was calculated based on the median λ value for each chromosome and subsequently, averaged across 22 chromosomes to obtain the overall λ.

SNP Annotation

LocusZoom [11] interactive plots of published GWAS results were used to examine known associations with IHS components using GWAS results from the follow studies: Diabetes Genetics Replication And Meta-analysis (DIAGRAM) 1000G type 2 diabetes (T2D) meta-analysis [12], Meta-Analysis of Glucose and Insulin-related traits Consortium (MAGIC) fasting glucose meta-analysis [13], Global Lipids Genetics Consortium (GLGC) total cholesterol, triglycerides, LDL cholesterol and HDL cholesterol meta-analysis [14], International Consortium for Blood Pressure (ICBP) systolic and diastolic blood pressure meta-analysis [15], and Genetic Investigation of Anthropometric Traits (GIANT) BMI meta-analysis, all ancestries [16,17].

**REFERENCES**

1. Manichaikul A, Mychaleckyj JC, Rich SS, Daly K, Sale M, Chen W-M. Robust relationship inference in genome-wide association studies. Bioinformatics. 2010 Nov 15;26(22):2867–73.

2. Klarin D, Damrauer SM, Cho K, Sun YV, Teslovich TM, Honerlaw J, et al. Genetics of blood lipids among ~300,000 multi-ethnic participants of the Million Veteran Program. Nat Genet. 2018 Nov;50(11):1514–23.

3. Price AL, Patterson NJ, Plenge RM, Weinblatt ME, Shadick NA, Reich D. Principal components analysis corrects for stratification in genome-wide association studies. Nat Genet. 2006 Aug;38(8):904–9.

4. 1000 Genomes Project Consortium, Auton A, Brooks LD, Durbin RM, Garrison EP, Kang HM, et al. A global reference for human genetic variation. Nature. 2015 Oct 1;526(7571):68–74.

5. Howie B, Fuchsberger C, Stephens M, Marchini J, Abecasis GR. Fast and accurate genotype imputation in genome-wide association studies through pre-phasing. Nat Genet. 2012 Jul 22;44(8):955–9.

6. Loh P-R, Palamara PF, Price AL. Fast and accurate long-range phasing in a UK Biobank cohort. Nat Genet. 2016 Jul;48(7):811–6.

7. Winkler TW, Day FR, Croteau-Chonka DC, Wood AR, Locke AE, Mägi R, et al. Quality control and conduct of genome-wide association meta-analyses. Nat Protoc. 2014 May;9(5):1192–212.

8. Cardon LR, Palmer LJ. Population stratification and spurious allelic association. Lancet. 2003 Feb 15;361(9357):598–604.

9. Marchini J, Cardon LR, Phillips MS, Donnelly P. The effects of human population structure on large genetic association studies. Nat Genet. 2004 May;36(5):512–7.

10. Campbell CD, Ogburn EL, Lunetta KL, Lyon HN, Freedman ML, Groop LC, et al. Demonstrating stratification in a European American population. Nat Genet. 2005 Aug;37(8):868–72.

11. Pruim RJ, Welch RP, Sanna S, Teslovich TM, Chines PS, Gliedt TP, et al. LocusZoom: regional visualization of genome-wide association scan results. Bioinformatics. 2010 Sep 15;26(18):2336–7.

12. Scott RA, Scott LJ, Mägi R, Marullo L, Gaulton KJ, Kaakinen M, et al. An Expanded Genome-Wide Association Study of Type 2 Diabetes in Europeans. Diabetes. 2017 Nov;66(11):2888–902.

13. Manning AK, Hivert M-F, Scott RA, Grimsby JL, Bouatia-Naji N, Chen H, et al. A genome-wide approach accounting for body mass index identifies genetic variants influencing fasting glycemic traits and insulin resistance. Nat Genet. 2012 May 13;44(6):659–69.

14. Willer CJ, Schmidt EM, Sengupta S, Peloso GM, Gustafsson S, Kanoni S, et al. Discovery and refinement of loci associated with lipid levels. Nat Genet. 2013 Nov;45(11):1274–83.

15. International Consortium for Blood Pressure Genome-Wide Association Studies, Ehret GB, Munroe PB, Rice KM, Bochud M, Johnson AD, et al. Genetic variants in novel pathways influence blood pressure and cardiovascular disease risk. Nature. 2011 Sep 11;478(7367):103–9.

16. Locke AE, Kahali B, Berndt SI, Justice AE, Pers TH, Day FR, et al. Genetic studies of body mass index yield new insights for obesity biology. Nature. 2015 Feb 12;518(7538):197–206.

17. Speliotes EK, Willer CJ, Berndt SI, Monda KL, Thorleifsson G, Jackson AU, et al. Association analyses of 249,796 individuals reveal 18 new loci associated with body mass index. Nat Genet. 2010 Nov;42(11):937–48.

**SUPPLEMENTAL FIGURES**

**Supplemental Figure 1: Ideal Health Score GWAS Meta-analysis QQ-Plot**

**
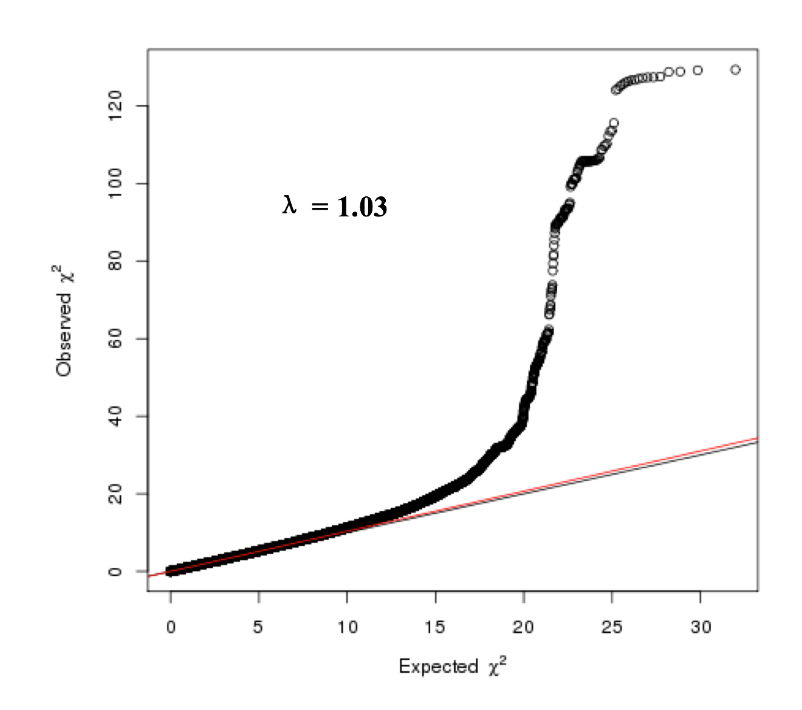
**

**Supplemental Figure 2: European Ideal Health Score GWAS Manhattan Plot**

**
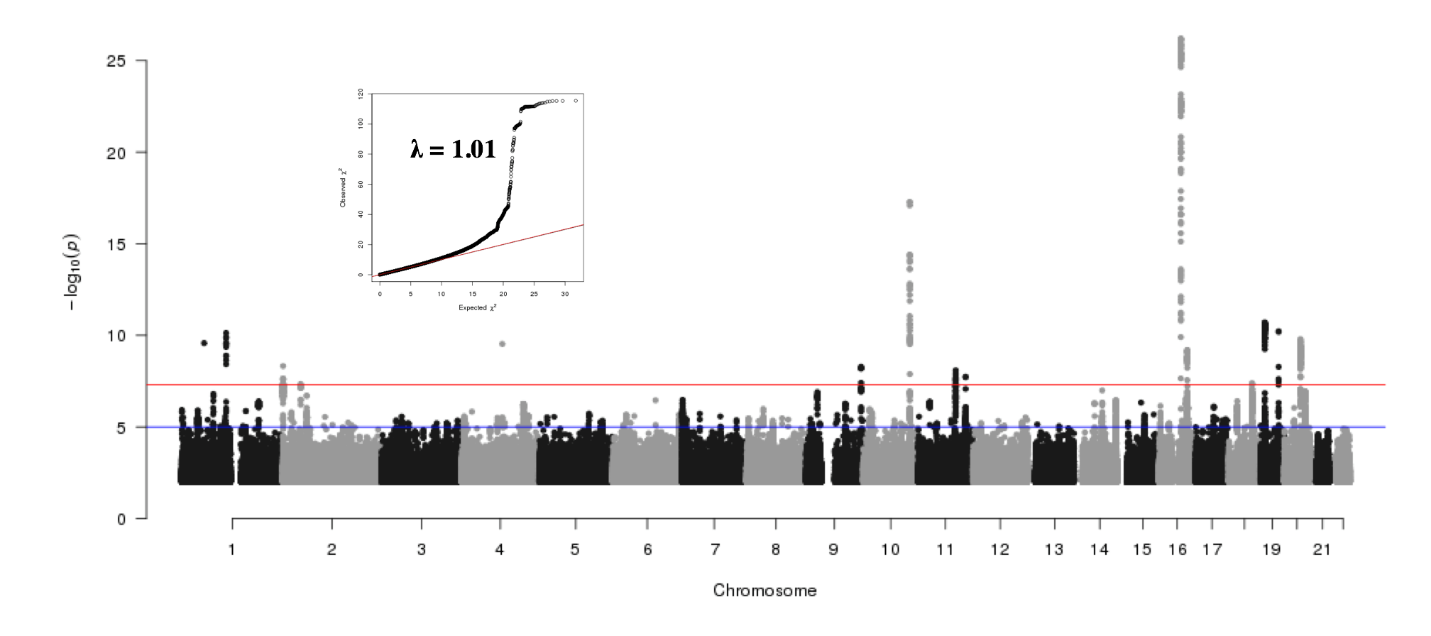
**

**Supplemental Figure 3: African Ideal Health Score GWAS Manhattan Plot**

**
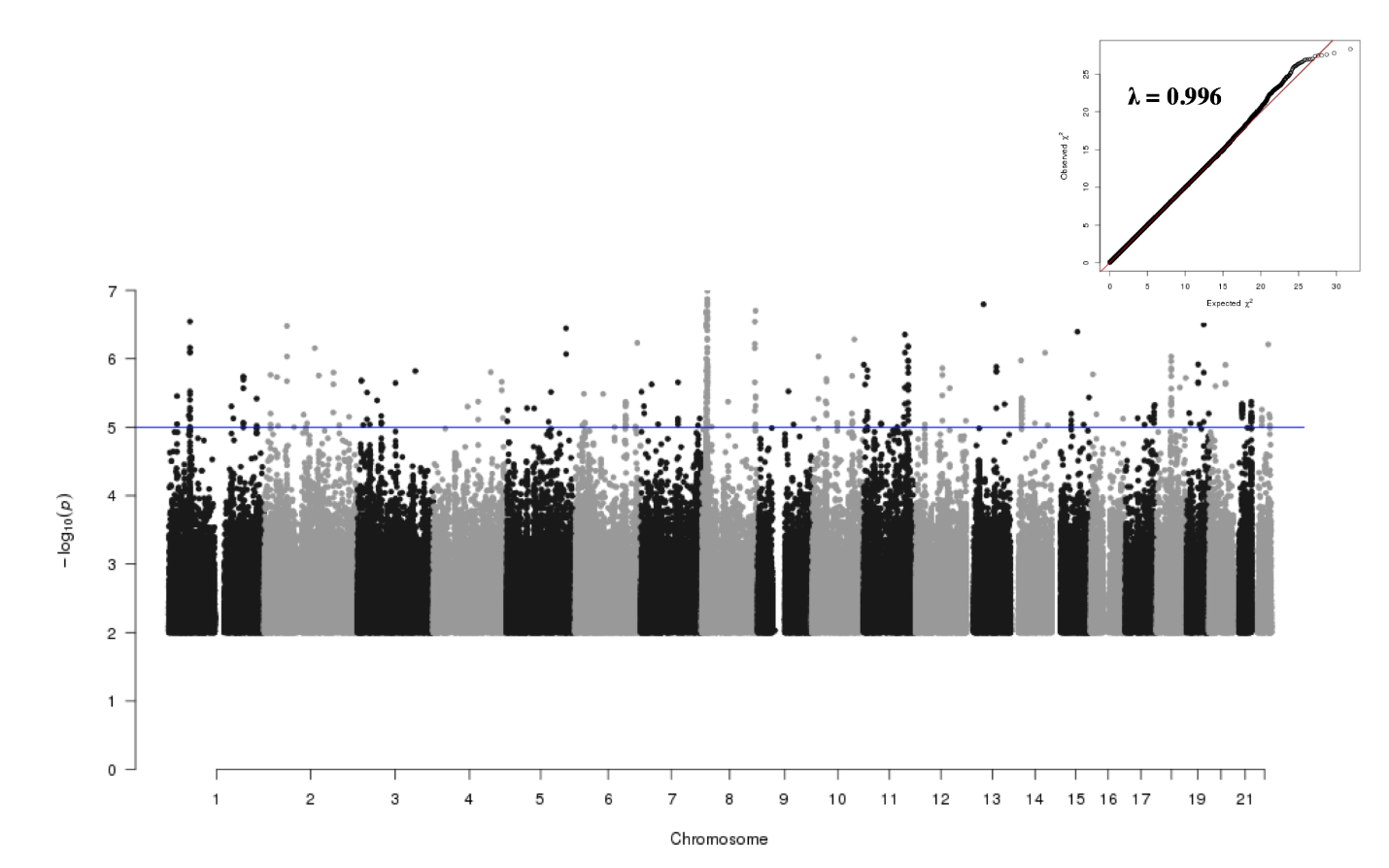
**

**Supplemental Figure 4: Hispanic Ideal Health Score GWAS Manhattan Plot**


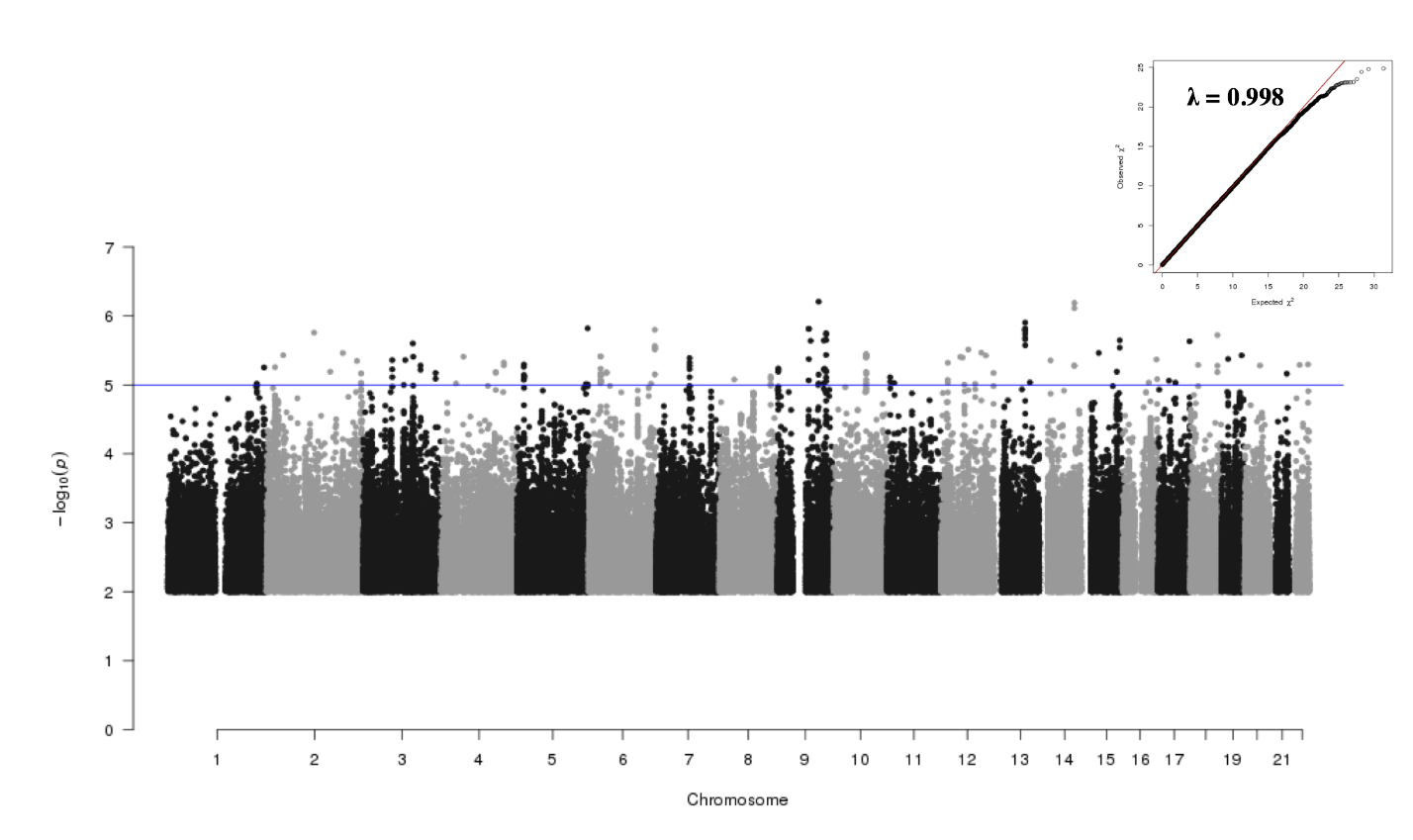


**Supplemental Figure 5. MVP African American PheWAS Manhattan Plot: Ideal Health Score Polygenic Risk Score vs Disease Phecode.** The negative log of the p-value is plotted for each of 882 disease phenotypes or “phecodes” in MVP. The horizontal red line indicates the statistically significant threshold (P < 5.67E-05). Each color represents a disease category as defined on the x-axis. Loci achieving p<1E-10 are annotated with the phecode description.


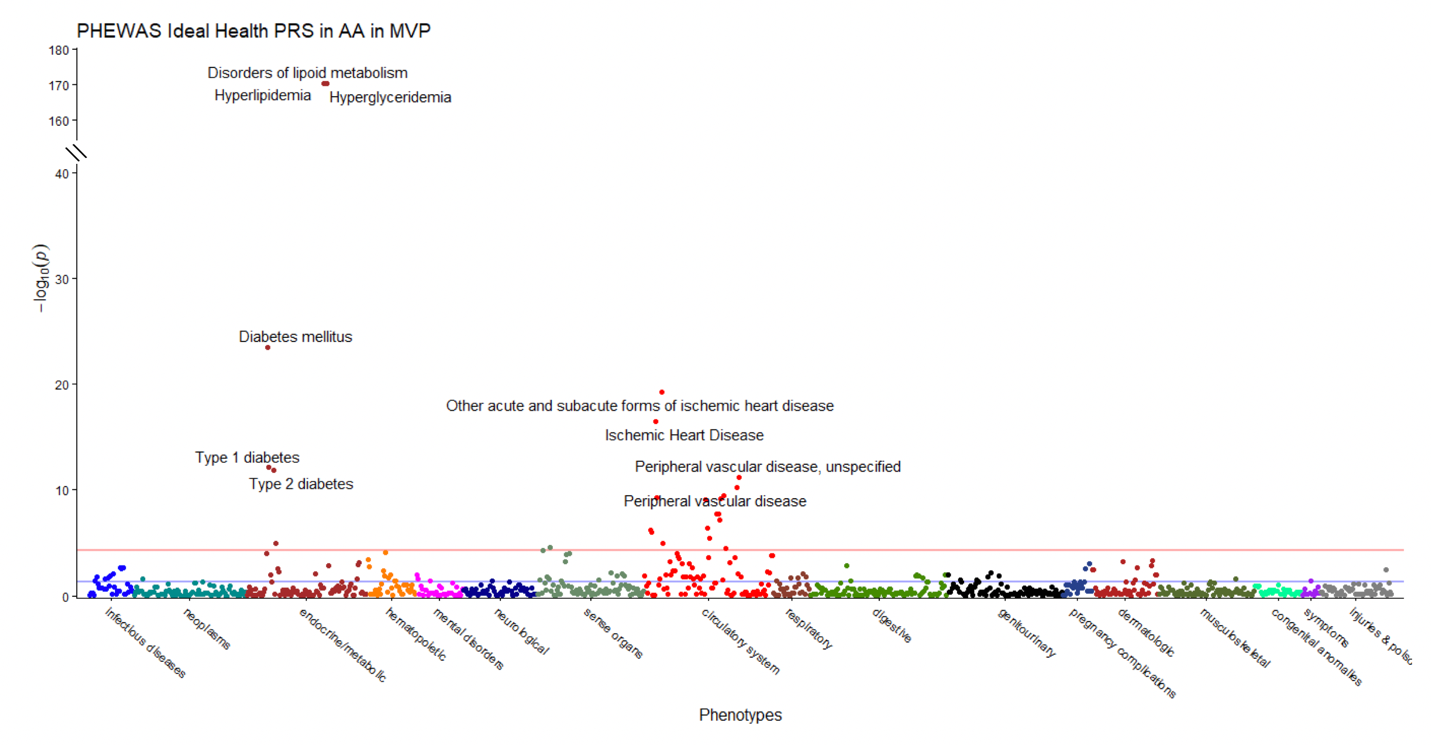


**Supplemental Figure 6. MVP Hispanics PheWAS Manhattan Plot: Ideal Health Score Polygenic Risk Score vs Disease Phecode.** The negative log of the p-value is plotted for each of 882 disease phenotypes or “phecodes” in MVP. The horizontal red line indicates the statistically significant threshold (P < 5.67E-05). Each color represents a disease category as defined on the x-axis. Loci achieving p<1E-5 are annotated with the phecode description.


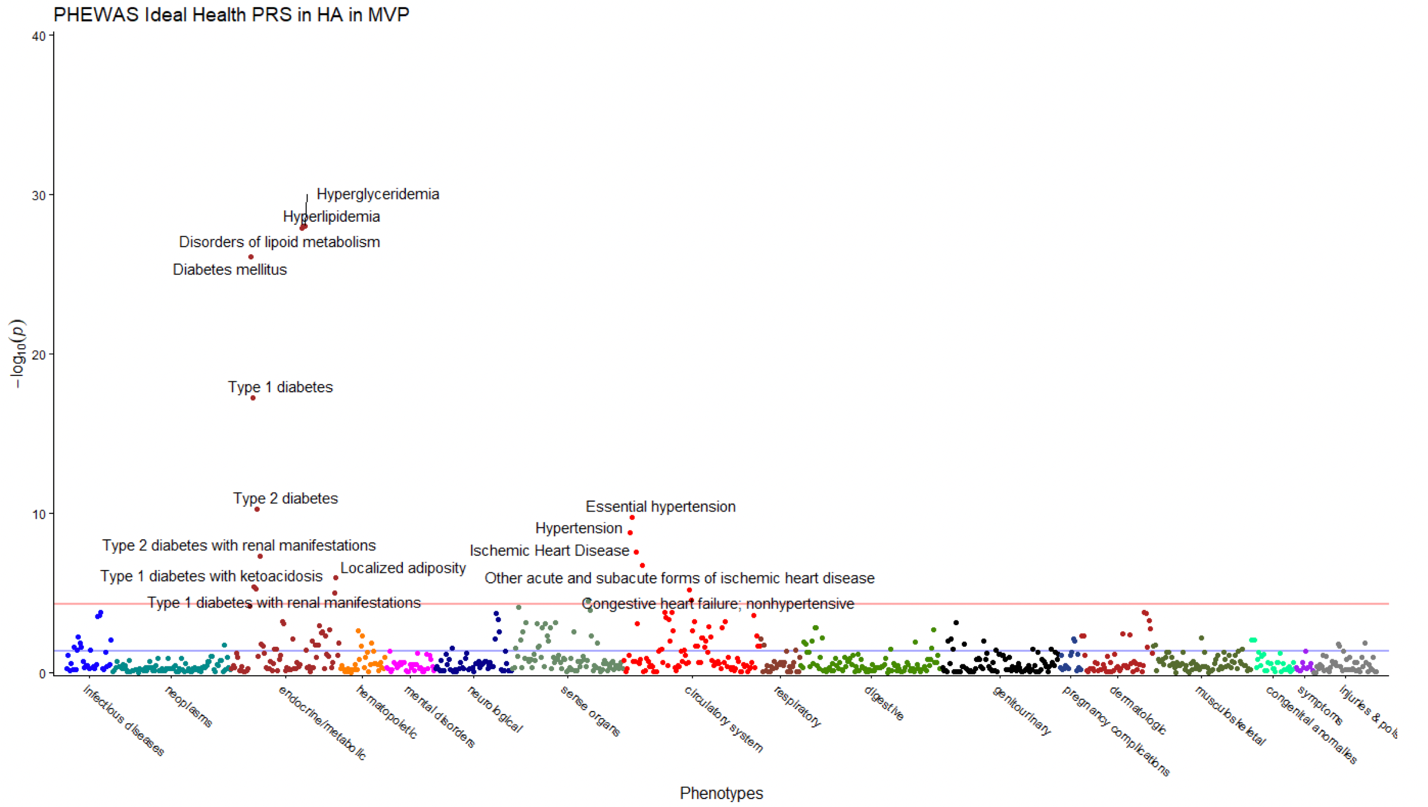


**Supplemental Figure 7. MVP European American PheWAS Manhattan Plot: Ideal Health Score Polygenic Risk Score vs Disease Phecode.** The negative log of the p-value is plotted for each of 882 disease phenotypes or “phecodes” in MVP. The horizontal red line indicates the statistically significant threshold (P < 5.67E-05). Each color represents a disease category as defined on the x-axis. Loci achieving p<1E-25 are annotated with the phecode description.

**
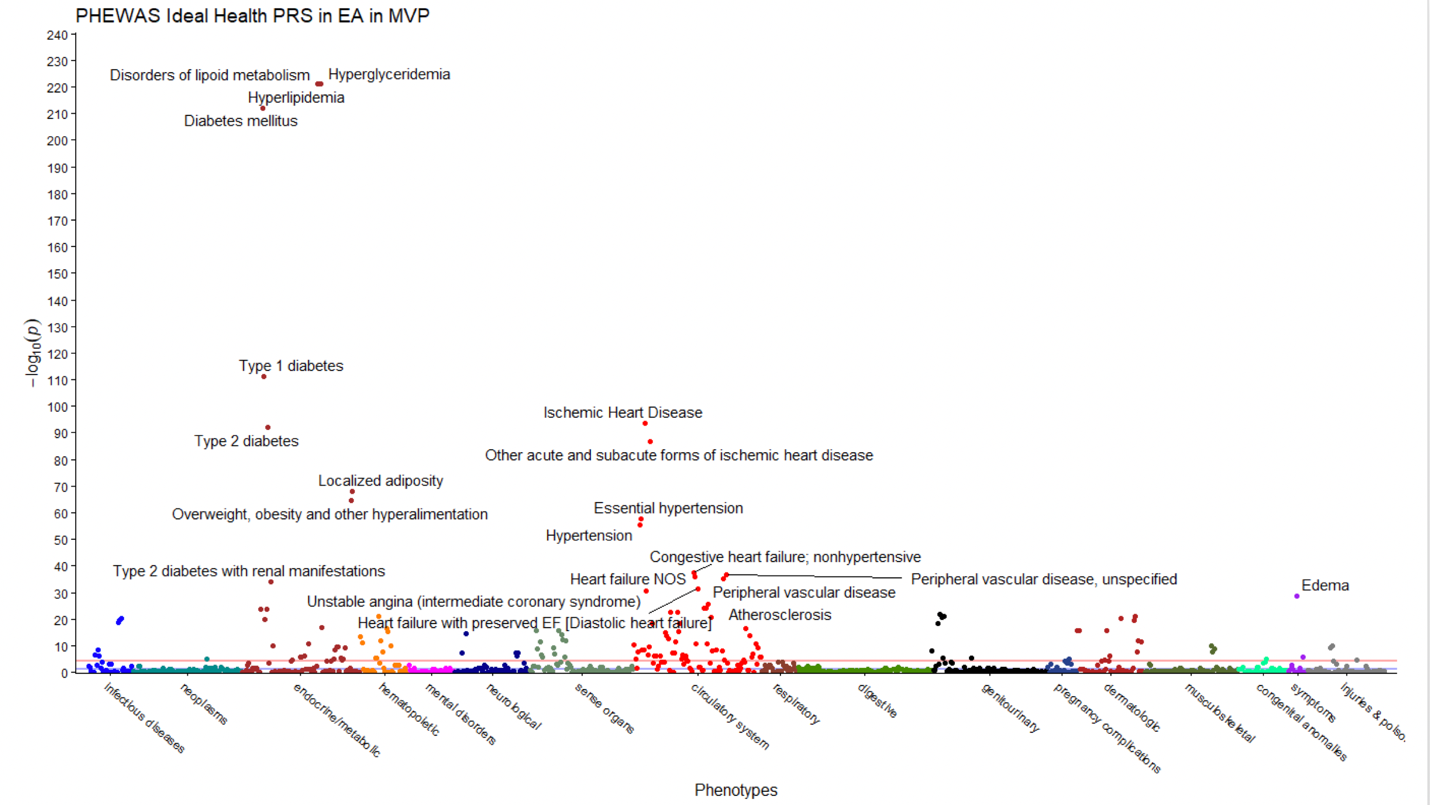
**

**Supplemental Figure 8. UKBB European American PheWAS Manhattan Plot: Ideal Health Score Polygenic Risk Score vs Disease Phecode.** The negative log of the p-value is plotted for each of 1,084 disease phenotypes or “phecodes” in MVP. The horizontal red line indicates the statistically significant threshold (P < 4.61E-05). Each color represents a disease category as defined on the x-axis. Loci achieving p<1E-11 are annotated with the phecode description.

**
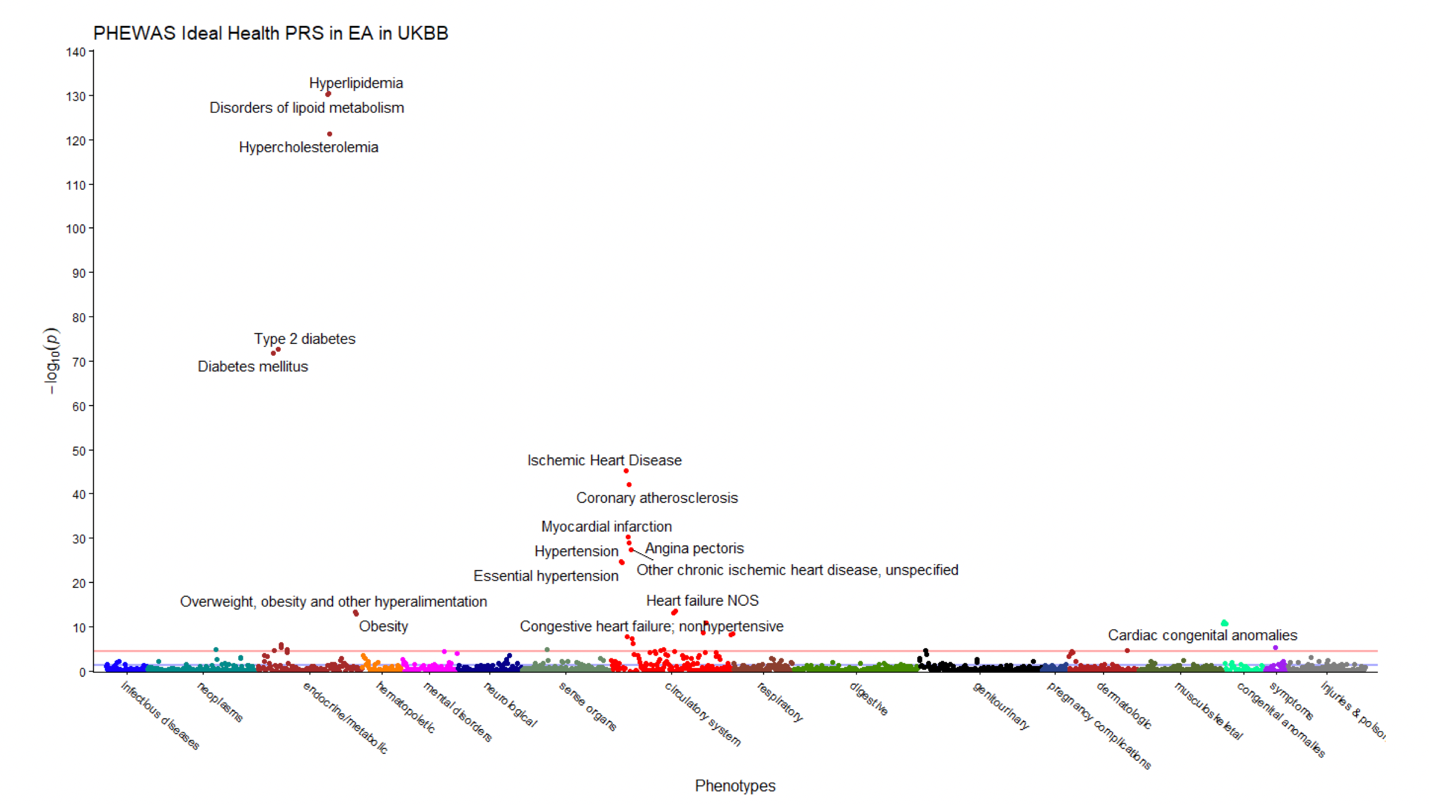
**
